# A Pooled Blood Genome-Wide Association Study of Hypertension in Sindhi Families: Results from the DISFIN Study

**DOI:** 10.3390/genes17030351

**Published:** 2026-03-22

**Authors:** Samika Kanaskar, Ashwini A. Patel, Manisha T. Jaisinghani, Kanchan V. Pipal, Mangesh Kanaskar, Manju Mamtani, Hemant Kulkarni

**Affiliations:** 1 Department of Public Health & Health Professions, University of Florida, Gainesville, FL 32611, USA; 2 Lata Medical Research Foundation, Nagpur 440022, India; 3 Precisely Software, Inc., Burlington, MA 01803, USA; 4 M&H Research, LLC., San Antonio, TX 78249, USA

**Keywords:** type 2 diabetes, genome-wide association study, ethnicity

## Abstract

Background: Hypertension is an important target for primordial prevention of complex, noncommunicable diseases, and its prevalence remains high across populations. The urban population in India is at a high risk of hypertension, but the genetic basis of hypertension in this population remains poorly understood. Methods: We conducted a pooled whole-blood genome-wide association study of 28 pools representing 1402 participants of the Diabetes In Sindhi Families In Nagpur (DISFIN) study, which enrolled families of probands with type 2 diabetes (T2D). Genotyping was done using Illumina’s Global Screening Array. Results: From a total of 608,550 single-nucleotide variants, 191 were found to be significantly associated with hypertension even after adjusting for metabolic comorbidities, batch effects, pooling error, kinship status, and pooling variation. These variants mapped to 180 well-characterized genes comprising 55 (31%) genes, and encode long noncoding RNAs (lncRNAs). Many of the genes significantly associated with hypertension (including 35% of the lncRNAs) have also been reported by other studies. However, we identified novel genes (*SBF2*, *ARHGAP12*, *EPAS1*, *CLEC16A*, and *LRPPRC*) to be associated with hypertension. The most significantly associated lncRNA gene was *FLYWCH*-*AS1*. Bioinformatic analyses indicated that these novel genes are likely to have functional importance in hypertension. Conclusions: Our study thus points to the potential candidate genes associated with hypertension in endogamous Sindhi families with T2D patients. The replicable and functional role of these candidate genes should be investigated in future studies.

## 1. Introduction

Hypertension continues to be a common primordial risk factor for several cardiometabolic conditions, including diabetes, cardiovascular diseases, and chronic kidney disease. The World Global Report on hypertension estimated that the prevalence of hypertension was 33% in the age group of 30–79 years. Further, only 54% of those with hypertension are diagnosed, 42% are receiving treatment, and only 21% successfully control hypertension [1]. Interestingly, essential hypertension—where the cause of hypertension is unknown—is known to be influenced in part by both genetic and environmental risk factors as well as by the interactions between genetic and environmental factors. Previous studies from various parts of the world have been elegantly summarized [2,3,4], revealing that the estimated heritability of hypertension ranges from 30% to 60%. A common approach to understanding the genetic basis of complex diseases such as hypertension is to conduct a genome-wide association study (GWAS), which aims to identify key genetic variants associated with this disease. To date, over 2000 genetic variants have been identified in diverse populations to be associated with hypertension [5]. However, despite the 708 non-interactive and 38 environment-interactive genetic variants detailed by Waken et al. [4], a comprehensive understanding of the genetic drivers of hypertension remains elusive.

The estimated prevalence of hypertension in India is high, 24% in males and 20% in females [6,7]. Recent studies from the INDIGENIUS Consortium have demonstrated that within different ethnic backgrounds in India, the heritability estimates for systolic and diastolic blood pressure traits range between 0.11 and 0.39 and 0.13–0.38, respectively, indicating a noticeable genetic component to blood pressure [8]. Despite this knowledge, the genetic and genomic studies of hypertension in India have been few and far between. These studies have attempted to quantify the association between genetic variants and blood pressure traits [9] as a part of a larger study, but dedicated Indian-population-specific GWAS studies on hypertension and blood pressure–related traits are currently lacking.

We conducted a genome-wide interrogation of genetic variants associated with the risk of hypertension using pooled whole blood. The participants from whom blood was collected were enrolled in the Diabetes In Sindhi Families In Nagpur (DISFIN) study [10]. The DISFIN Study was designed with a focus on the genetics of type 2 diabetes, but the prevalence of essential hypertension in this study was as high as 53%, and thus provided us with an opportunity to conduct a genome-wide association study of hypertension as well. In the population studied, there was a large co-occurrence of type 2 diabetes, dyslipidemia, and obesity. Since our work was constrained by project costs, we conducted a pooled GWAS study of hypertension. Here, we report the results of our study on the genetic association of hypertension in the urban Indian Sindhi families.

## 2. Materials and Methods

### 2.1. Study Participants

For this study, we used the clinical and genetic data collected during the DISFIN study. The enrollment protocol used, the eligibility criteria, and the overall study design have been described elsewhere [10]. Participants were enrolled and blood samples collected during a year-long interval starting on 1 March 2017. For pedigree construction, we enrolled endogamous Sindhi families with ≥1 case of type 2 diabetes per family. Moreover, participants resided in the study area (Jaripatka, Mecosabag, and Khamla areas of Nagpur, which are high-Sindhi density areas); self-reported Sindhi ethnicity and age ≥20 years. Exclusion criteria were pregnant or lactating women and patients with type 1 diabetes. After administering semi-structured interviews and clinical examination, a trained phlebotomist collected blood samples for laboratory procedures.

### 2.2. Definitions of Metabolic Conditions

Our study was designed to conduct a genome-wide interrogation in the context of hypertension. Hypertension was defined as self-reported hypertension or currently on anti-hypertensives or systolic blood pressure ≥ 130 mmHg or diastolic blood pressure ≥ 85 mmHg [11]. Type 2 diabetes was defined as one or more of the following: self-reported diabetes; currently on anti-diabetics; fasting plasma glucose ≥ 126 mg/dL; random blood glucose ≥ 200 mg/dL; or HbA1c concentration ≥ 6.5% [12]. We defined central obesity based on waist circumference cutoffs for the Indian population [13] of ≥90 cm for males and ≥85 cm for females. Lastly, dyslipidemia was defined [14] as presence of any of the following: serum triglycerides ≥ 150 mg/dL or serum high density lipoproteins < 40 mg/dL (males) or <50 mg/dL (females).

### 2.3. Pool Definitions for GWAS

The technique of pooled blood GWAS is now well established as an acceptable alternative to individual genome-wide genotyping [15]. We previously conducted a pooled blood, genome-wide investigation for type 2 diabetes and have extensively described the definitions of whole blood pools used in the study [16]. Using the presence or absence of hypertension, central obesity, type 2 diabetes, and dyslipidemia, we first generated a total of 16 combinations and then reduced the number of pools to 14 (by collapsing those pool categories that had a frequency < 1%). Pools were run in duplicates. The pool construction, DNA extraction, and genotyping protocols have been described previously [16]. We used the Infinium Global Screening Array (GSA, Illumina, Inc., San Diego, CA, USA) for genotyping. The methods used for blood sample collection, storage, extraction of DNA, and genotyping have been described elsewhere [16].

### 2.4. Statistical Analyses

The GSA array returned allele frequencies for each genetic marker for all 28 pools. Since we wished to arrive at robust, independent associations of the allele frequency with hypertension, we adjusted for the following sets of covariates: comorbidities, assay characteristics (batch effects, intra-replicate correlation, within-pool genetic similarity), and the random effects across pools. We used the mixed effects logistic regression, which yielded the Wald test T statistic to test the association of a variant with the risk of hypertension. Specifically, the following regression model was used to estimate the association:

logit(hypertension) = *β*_0_ + *β*_s_ B + (*β*_1_ C1 + *β*_2_ C2 + *β*_3_ C3) + (*β*_b_ A1 + *β*_r_ A2 + *β*_k_ A3) + RE(P)

where hypertension is an indicator variable for presence of hypertension; B is the B allele frequency; C1–C3 are comorbidities (type 2 diabetes, central obesity and dyslipidemia); A1–A3 represent assay characteristics: A1 is the chip identifier, A2 is the replicate identifier, and A3 the within pool degree of genetic similarity; and, lastly RE(P) captures the random effects across the study pools. The regression coefficients in the equation were used to quantify the differential influence of the B allele frequency (*β*_s_), influence of comorbidities (*β*_1_–*β*_3_), batch effect (*β*_b_), pooling error (*β*_r_), and kinship effect (*β*_k_). All the models were weighted by the pool frequency. To account for multiple comparisons, we used an adjustment of the false discovery rate using the Benjamini–Hochberg method [17]. All the analyses used R; the Manhattan and QQ plots were created using the qqman library [18] in R (version 4.5.2). All R scripts used in this study are described in an annotated fashion in Supplementary Notes S1–S3. Pooling error was estimated per MacGregor et al. [19].

### 2.5. Functional Role of Variants and Genes

For annotation of the variants and for a comprehensive, genomic understanding of their role in health and disease, we used the SNPAnnotator R package [20]. In addition to the in-built abilities of SNPAnnotator, we also used the g:Profiler online tool (https://biit.cs.ut.ee/gprofiler/gost, accessed on 29 January 2026) to conduct gene set enrichment analyses. The results from both SNPAnnotator and g:Profiler were reported as *p*-values after controlling the false discovery rate (FDRp). Deleteriousness of variants was estimated using the Combined Annotation Dependent Depletion (CADD, https://cadd.gs.washington.edu, accessed on 20 January 2026) score.

## 3. Results

### 3.1. Study Participants and Pools

This study represents a secondary analysis of the data derived from the DISFIN study. As described elsewhere, the study included a total of 1444 participants representing 112 endogamous Sindhi families in Nagpur, Maharashtra, India. Our study combined the whole-blood samples with clinical data (n = 1402) collected into 28 pools based on the presence or absence of four dichotomous clinical traits: hypertension, type 2 diabetes, dyslipidemia, and obesity. For this study, two pools were derived from the 28 pools to compare the presence or absence of hypertension as the trait of interest. The pool with hypertension represented whole-blood pooling of 742 (52.92%) participants. Using the same inclusion criteria for genetic variants as described previously [16] we included a total of 608,550 autosomal single-nucleotide polymorphisms (SNPs) with a minor allele frequency >0.1. It is noteworthy that the genotyping error (as measured using the GenTrain score) and the pooling error estimates were acceptable [16].

### 3.2. Heritability of Blood Pressure–Related Traits

We first estimated the heritability of blood pressure traits in the study population. We found that the heritability estimates for the continuous traits: systolic blood pressure, diastolic blood pressure, pulse pressure, and mean arterial pressure were 0.24 (SE 0.08, *p* = 0.0010), 0.31 (SE 0.08, *p* = 0.0004), 0.18 (SE 0.08, *p* = 0.0106), and 0.15 (SE 0.08, *p* = 0.0149), respectively. The heritability of hypertension (estimated using the liability threshold approach for a dichotomous trait) was 0.44 (SE 0.14, *p* = 0.0003), indicating that all traits studied here in the context of blood pressure showed a statistically significant and clinically meaningful heritability. For the genome-wide association study, we focused on the dichotomous trait of hypertension.

### 3.3. Pooled GWAS Results at the Level of Variants

**Figure 1:**
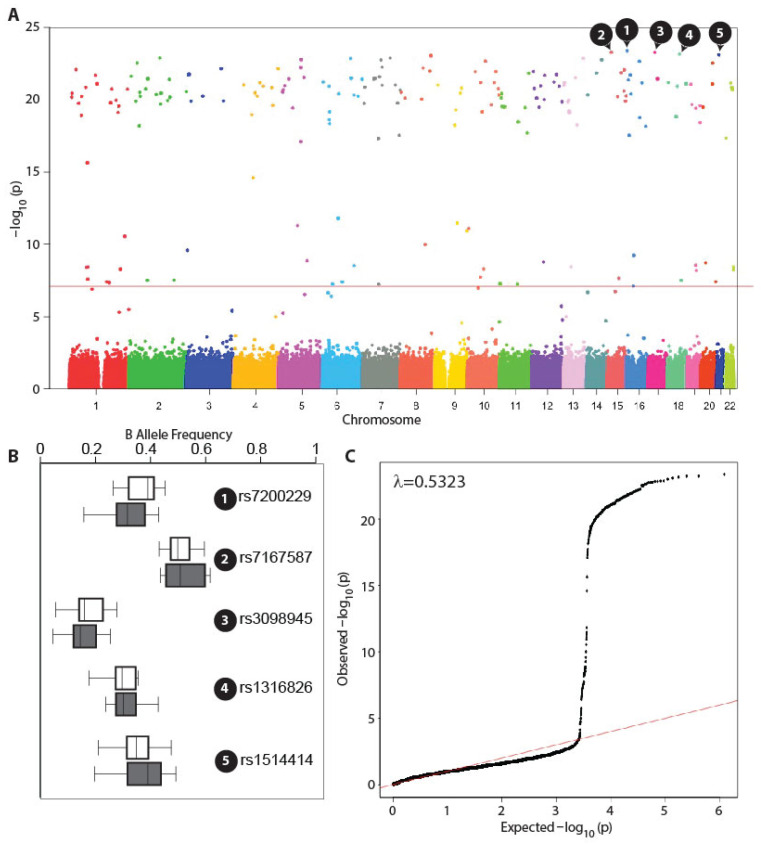
Results of the whole-blood, pooled genome-wide association study of hypertension. (**A**) Manhattan plot. The points show the log-transformed adjusted and corrected p-values, which are statistically significant. The five topmost significant associations are numbered as 1 through 5. The red line indicates global type I error rate of 0.05 (**B**) Box plots for the distribution of the top five significant SNP markers. Open and closed boxes are for pools without and with hypertension, respectively. The numbers indicated in black circles correspond to those in panel A. (**C**) QQ plot. The plot shows the quantile relationship between the observed and expected *p*-value distribution. Shown at the top is the genomic inflation factor (λ). The red line shows the expected distribution.

Of the 608,550 autosomal markers studied here, we found that a total of 191 variants were significantly associated with hypertension even after adjusting for the covariates listed in Section 2 and the multiple comparisons alluded to earlier. The fully annotated description of the significantly associated 191 variants is provided in Supplementary Table S1. The genome-wide association pattern observed in the present study is shown in Figure 1A. Also, as shown in the QQ plot depicted in Figure 1C, we found that the genomic inflation factor (λ) was below unity, indicating that there was negligible genomic inflation during genotypic assays. Of note, only nine variants were associated with a CADD score between 10 and 20, while three variants (rs28933396, rs74740987, and rs10075131) were associated with a CADD score above 20. This indicated that with respect to deleteriousness, the majority of the variants were benign.

**Figure 2:**
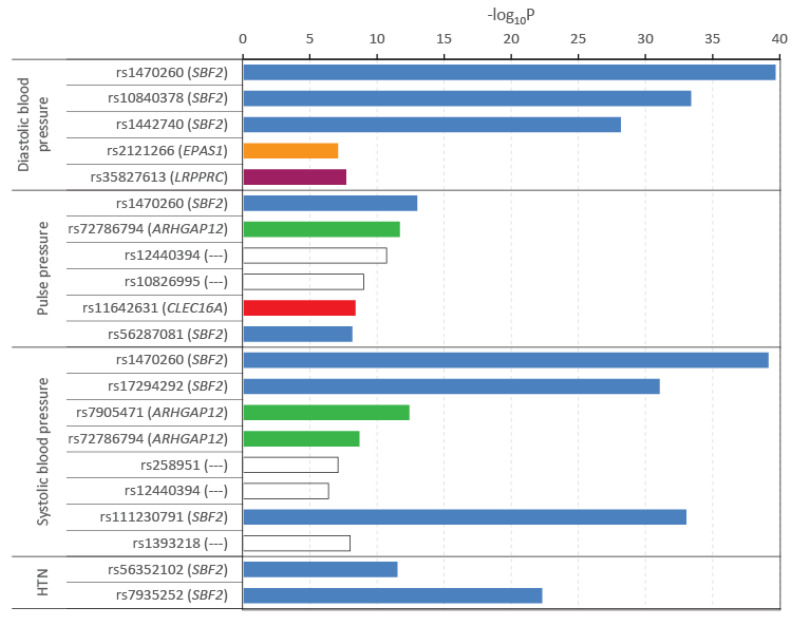
Association of significant variants with blood pressure–related traits from published studies in GWASCatalog. Names of the variants (gene) are shown on the vertical axis, while the horizontal bars represent the log-transformed *p*-value. Colors of the bars represent different genes—blue, *SBF*; green, *ARHGAP12*; orange, *EPAS1*; brown, *LRPPRC*; and red, *CLEC16A*. Hollow bars represent variants from intergenic regions not mapping to any gene. ‘---’; does not map to any gene.

The top five most significant markers (highlighted in Figure 1A,B) were following single-nucleotide polymorphisms: rs7200229, rs7167587, rs3098945, rs1316826, and rs1514414. Queries run through the SNPAnnotator package identified two of these five polymorphisms—rs7200229 and rs3098945. The rs7200229 SNP is a non-coding exon variant associated with the *FLYWCH1-AS1* gene on chromosome 16. The rs3098945 polymorphism is an intronic variant in the *ANKRD13B* gene on chromosome 17. Interestingly, 23 of the 191 variants have been previously reported by other genome-wide association studies (as queried against the Human Phenotype Ontology database), indicating that our study could replicate several of the known associations in the context of GWAS. The subset of variants found in the GWASCatalog to be associated with blood pressure–related traits (shown in Figure 2) was consistent with this finding. The observed network of SNPs and disease association from the GWASCatalog is shown in Supplementary Figure S1.

### 3.4. Pooled GWAS Results at the Level of Genes

The top 191 significantly associated SNPs mapped to 149 known and named genes (Supplementary Table S2). Of the 191 variants queried, the SNPAnnotator module could map 180 genes that included a total of 107 (59.44%) protein-coding genes, 55 (30.56%) long noncoding RNA (lncRNA) genes, 1 small Cajal-body specific RNA gene, 1 small nuclear RNA gene, and 16 pseudogenes. Of the 55 lncRNA genes, 37 (67.27%) were intergenic, 8 (14.54%) were intronic, 6 (10.91%) were antisense, and 4 (7.28%) were sense lncRNAs. A full list of the variants associated with the lncRNAs is provided in Supplementary Table S3. Literature search revealed that 19 (34.54%) of the lncRNAs listed in Supplementary Table S3 have been previously reported to be associated with blood pressure–related traits.

When the list of 149 named genes was queried against the Human Phenotype Ontology (HPO) terms, a total of 33 terms were significantly (FDR-corrected *p* < 0.05) associated with the list (Supplementary Table S4). Strikingly, the list contained the following terms: blood pressure (FDRp = 0.0132), systolic blood pressure (FDRp = 0.0194), diabetes mellitus (FDRp = 0.0120), triglyceride measurement (FDRp = 0.0440), and body weight measurement (FDRp = 2.61 × 10^−5^). These results affirmed a biological explanation and the strong plausibility of metabolic function in accounting for the observed association pattern. The genes associated with the terms blood pressure and systolic blood pressure included: *GALNT18*, *SBF2*, *VIPR2*, *TENM4*, *SHROOM3*, *DUSP16*, *ZNF609*, *DGKH*, *ACMSD*, *GRM7*, *ZNF98*, *AGBL4*, *SIK3*, *CDH18*, *ALK*, *ZFPM2*, *RBFOX1*, *FTO*, *FGD4*, *PAFAH1B2*, *TRPC4*, *CSMD1*, and *LRP2*. Comparatively, when gene enrichment analyses were conducted for the gene ontology terms using the g:Profiler tool, we found (Supplementary Figure S2) that six terms were significantly associated with the gene set. These were: ion binding (FDRp = 0.0313), transmembrane transporter binding (FDRp = 0.0417), anatomical structure development (FDRp = 0.0023), biological regulation (FDRp = 0.0126), axon (FDRp = 0.0002), and juxtaparanode region of axon (FDRp = 0.0165).

In addition, novel associations found in relation to the topmost significant variants revealed some interesting patterns. For example, well-characterized genes related to the top 20 significant variants were associated with the gene *FLYWCH1-AS1* (rs7200229), *ANKRD13B* (rs3098945), *RNU6-976P* (rs17258345), *MAGI2* (rs12665877), *DUSP29* (rs755228), *COX6CP2* (rs8183309), *MTHFD2P5* (rs7457005), and *SDC2* (rs2008026). Further, some long non-coding genes included in this list were: *ENSG00000294624*, *LINC01320*, and *ENSG00000249776*. Indeed, results from Figure 2 indicated that variants in the genes *SBF2*, *ARHGAP12*, *EPAS1*, and *CLEC16A* were strongly associated with blood pressure–related traits in published GWAS studies. There were seven distinct variants in or around the *SBF2* gene, which were associated with one or more pressure–related traits, making it a potential determinant of the risk of hypertension in the study population.

## 4. Discussion

Our study made the following critical observations. First, we found a striking concurrence of type 2 diabetes, prediabetes, and hypertension in the urban Sindhi families. Second, we observed that whole-blood pooling and genotyping were able to identify interesting patterns of genetic variants that revealed known as well as novel genome-wide associations. Third, there was a specificity of association of the genetic variants with hypertension, such that several genomic hits observed by us have been reported by other genome-wide association studies previously. Fourth, we identified new variants and genes that were associated with hypertension in the study population. For example, the multipronged association of the *SBF2* gene variants, the links between *FLYWCH1-AS1* and *ANKRD13B* gene variants and hypertension, and the identification of several long noncoding RNA genes as potential markers of hypertension have yielded additional insights into hypertension pathophysiology. Lastly, the data presented here have not been previously described in the context of the urban Sindhi population studies.

The findings of our study need to be considered in the light of the increasing burden of hypertension in India. The prevalence of hypertension in our study was alarmingly high (>50%). This prevalence is not reflective of the general population prevalence because of at least two factors. First, the study participants were ascertained for the presence of at least one known patient of type 2 diabetes in the family. Since hypertension is a risk factor for type 2 diabetes, a higher proportion of study participants (as compared to the general population) is expected to have hypertension. In the 2015-16 data from the National Family Health Survey in India, the prevalence of hypertension in diabetic individuals was estimated to be 37% [21]. Second, this is a family study and therefore heritable traits are likely to cluster frequently in the study sample—a situation that can masquerade as a high prevalence rate. Our study design precludes the use of available methods for family-based designs to estimate the population prevalence of disease. Nevertheless, the estimated prevalence of hypertension in the study participants is indicative of a high prevalence of hypertension in the Indian urban Sindhi population. For example, the well-conducted and nationally representative ICMR-INDIAB study estimated the prevalence of hypertension to be 35.5% in India [22]. Similarly, the Indian Society of Hypertension estimated the prevalence of hypertension to be 21% and 24%, and the prevalence of pre-hypertension to be 39% and 49% in women and men, respectively [23]. Together, our study findings gain importance in the light of the increasing prevalence of hypertension in general and in individuals with metabolic comorbidities in particular. The novelty of our findings is further enhanced by the fact that, to our knowledge, this is the first study documenting a high prevalence of hypertension in the ethnically endogamous group of urban Sindhis in India.

Large international efforts to understand the genetic determinants have resulted in several important GWAS studies and meta-analyses; however, the majority of these have studied European, African, or East Asian populations. These results have been elegantly summarized first by Franceschini et al. [24] and then by Wang and Wang [25]. For example, Franceschini et al. [24] used 19 African ancestry cohorts totaling 29,378 individuals and 7 non-African ancestry cohorts totaling 11,763 individuals to meta-analytically synthesize genetic associations with hypertension in the development and replication phases, respectively. Keaton et al. [5] studied one million individuals of European ancestry and found 2103 variants to be significantly associated with hypertension. Recent large studies on East Asian populations include those reported by Pozarickij et al. [26] and Li et al. [27]. These studies have generally used systolic blood pressure, diastolic blood pressure, pulse pressure, and arterial pressure as the traits of interest. These studies and other reviews [28] have consistently shown that hypertension is a significantly heritable trait, polygenic in nature, and has population-specificity of genetic association. Interestingly, however, very little data exists on the genetic association of hypertension in South Asians in general. Specifically, for the Indian ethnically endogamous population group, which is the focus of the current investigation, this is the first such investigation.

We found variants related to five genes known to be associated with hypertension through well-recognized biological mechanisms. Of these five susceptibility genes, variants in and around the *SBF2* gene were the most common. The *SBF2* gene (also called the *MTMR13* gene) encodes a protein involved in phosphoinositide signaling [29]. This mechanism has been strongly implicated in the development of hypertension (https://maayanlab.cloud/Harmonizome/gene_set/Hypertension/GWAS+Catalog+SNP-Phenotype+Associations+2025 accessed on 11 February, 2026, [30]). On the other hand, the *ARHGAP12* gene interacts with the With No Lysine (K) pathway (WNK pathway), which is a key regulator of blood pressure [31]. Similarly, endothelial Epas1 (the protein product of the *EPAS1* gene) has been implicated in renal damage, resulting in focal segmental glomerulosclerosis that manifests as hypertension [32]. Similarly, the *CLEC16A* gene is known to be involved in mitochondrial activity regulation that exercises renal control of blood pressure [33] as well as vascular stiffness [34]. Lastly, the *LRPPRC* gene also participates in vascular tone control and oxidative stress through mitochondrial pathways [35,36] and can, thus, influence the risk of hypertension indirectly.

We also found some additional interesting genes as susceptibility loci for hypertension in the Sindhi families. Of note, the *FLYCH1-AS1*, which is a component of the Wnt signaling pathway, is a long noncoding RNA. Derangements in Wnt signaling (which *FLYWCH1* modulates and *WNT2* participates in) cause vascular smooth muscle remodeling that may underlie hypertension and vascular diseases [37,38]. Similarly, the *ANKRD13B* gene has been found in genome-wide association studies to be linked with both coronary artery disease and blood pressure traits [39]. The *MAGI2* gene is primarily involved in synaptic functions and has been associated with blood pressure regulation in a large-scale study on 564,680 participants from diverse populations [40]. *SDC2*, which encodes syndecan-2, is functionally known to play a part in maintaining vascular endothelial integrity. Its close family member, syndecan-4, has been implicated in blood pressure regulation [41].

Two more observations merit a mention. First, we found several lncRNA genes (approximately 31% of the significant genes) to be associated with hypertension. It is noteworthy that a recent transcriptome-wide association study [42] found that 30 transcripts of lncRNA (especially related to the *UCP2* gene) were significantly associated with hypertension. Jiang and Ning [43] have summarized the potential of lncRNAs as mediators of blood pressure. Currently, the exact mechanism underlying the contribution is unknown and a matter of scientific interest [44]. Our study found several interesting lncRNAs that need further replicative confirmation and functional relevance assessment. Second, on the other extreme, we found that pseudogenes such as *RNU-976P*, *COX6CP2*, and *MTHFD2P5* were associated with hypertension, but the functional role of such associations is unknown.

In addition to the previously described advantages and strengths of this pooled GWAS approach [16] our study has some limitations. First, the concept of whole-blood pooled GWAS is predicated on the assumption that the allele frequencies are faithfully captured by pooling. In the absence of individual-level GWAS data, the veracity of this assumption cannot be commented upon. It should also be noted that pooled GWAS can be a good screening tool rather than evidence of a definitive genetic association. Therefore, whether the results of this study will hold if compared to an individual-level GWAS on the same participants cannot be inferred and should be considered in future studies. Second, the lack of data on individual genotyping also limits our ability to account for potential population stratification. Considering the ethnically endogamous disposition of the study participants, we do not anticipate a high degree of population stratification; however, without individual genotyping, direct accounting for population stratification could not be undertaken. Third, our study did not have a replication cohort. Considering the endogamous and related nature of the study participants, it is practically difficult to find or design a similar validation cohort. Further, to our knowledge, GWAS data on such a cohort are not available. Thus, generalization of the observed associations is not possible, and the lack of a validation cohort remains a limitation of the study. Fourth, all associations observed in this study are statistical, but the biological explanation for the functional role of the susceptible loci identified in this study is currently not available and cannot be inferred. Whereas future studies are needed to understand the functional role of genes and variants, we also compared the observed associations with those reported in well-characterized repositories and in published literature. Fifth, since the study enrolled patients with type 2 diabetes and their families, the results of the study should not be generalized to other populations. Sixth, the generalizability of our findings is also limited by the ethnic group enrolled and the geographic location of the study. Seventh, the clinical significance of the observed associations also remains unknown.

## 5. Conclusions

Notwithstanding these limitations, we conclude that the prevalence of hypertension in the endogamous Sindhi families studied here was high (52%) and this trait was highly heritable (h^2^r = 0.44). Our pooled, whole-blood GWAS for hypertension in the families of type 2 diabetes (T2D) patients uncovered interesting candidate genes for future investigations. We identified several significant variants and genes that have been reported by other studies previously. Notably, nearly 31% of the genes identified in this study were related to lncRNAs, which are being increasingly recognized as potential biomarkers of hypertension. Whether the genomic hits identified in this study are population-specific, whether there is a functional explanation for the role of these genomic variants in hypertension, and whether some of the identified lncRNAs can be considered biomarkers in the study population are important questions for future research.

## Data Availability

Lata Medical Research Foundation’s Institutional Ethics Committee (LMRF-IEC) does not allow public data sharing to avoid potential identification. If data is requested for verification of results, we will seek permission from the LMRF-IEC before the requested data can be released. For further clarification of the LMRF-IEC’s data access policy as well as for data access requests, please contact: Dr. Prabir Kumar Das, Member Secretary, Institutional Ethics Committee, Lata Medical Research Foundation, Kinkine Kutir, Vasant Nagar, Nagpur—440022, Ph. No. 91-8805023450, Email: prabir_das23@rediffmail.com.

## Supplementary Materials

The following supporting information can be downloaded at: https://www.mdpi.com/article/10.3390/genes17030351/s1, Supplementary Table S1: Annotated list of the significantly associated variants with hypertension in the DISFIN study participants; Supplementary Table S2: Annotation of the 149 named genes related to the 191 significantly associated variants; Supplementary Table S3: Long noncoding RNA genes associated with hypertension in the DISFIN Study; Supplementary Table S4: Human Phenotype Ontology terms related to significantly associated genes in the DISFIN Study; Supplementary Figure S1: Variant-phenotype network using data from GWASCatalog; Supplementary Figure S2: Gene set enrichment analyses using the g:Profiler tool; Supplementary Note S1: R code for conducting GWAS; Supplementary Note S2: R code for adjusting *p*-values; Supplementary Note S3: R code for creating plots and References [45,46,47,48,49,50,51,52,53] are cited in Supplementary Materials.
