# Supplementary material for: A Pooled Blood Genome-Wide Association Study of Hypertension in Sindhi Families: Results from the DISFIN Study
Source: Genes (Basel). 2026 Mar 22;17(3):351. doi: 10.3390/genes17030351 (PMC13026758; doi:10.3390/genes17030351)
Supplement: Supplementary file 1 [file genes-17-00351-s001.zip › genes-4217264-supplementary.pdf]

Supplementary Materials for

A Pooled Blood Genome-Wide Association Study of Hypertension in Sindhi Families: Results from the DISFIN Study

Samika Kanaskar [1,†], Ashwini A. Patel [2,†], Manisha T. Jaisinghani [2], Kanchan V. Pipal [2], Mangesh Kanaskar [3], Manju Mamtani [2,4] and Hemant Kulkarni [2,4,*]

[1] Department of Public Health & Health Professions, University of Florida, Gainesville, FL 32611, USA
[2] Lata Medical Research Foundation, Nagpur 440022, India
[3] Precisely Software, Inc., Burlington, MA 01803, USA
[4] M&H Research, LLC., San Antonio, TX 78249, USA
[*] Correspondence: hemant.kulkarni@mnhresearch.com
[†] These authors contributed equally to this work.

TABLE OF CONTENTS

Section	Pages
**Supplementary Tables**	**3-36**
Supplementary Table S1. Annotated list of the significantly associated variants with hypertension in the DISFIN study participants	3-8
Supplementary Table S2. Annotation of the 149 named genes related to the 191 significantly associated variants	9-26
Supplementary Table S3. Long noncoding RNA genes associated with hypertension in the DISFIN Study	27-30
Supplementary Table S4. Human Phenotype Ontology terms related to significantly associated genes in the DISFIN Study	31-36
**Supplementary Figures**	**37-38**
Supplementary Figure S1. Variant-phenotype network using data from GWASCatalog	37
Supplementary Figure S2. Gene set enrichment analyses using the g:Profiler tool	38
**Supplementary Notes**	**39-43**
Supplementary Note S1: R code for conducting GWAS	39-40
Supplementary Note S2: R code for adjusting p-values	41
Supplementary Note S3: R code for creating plots	42-43
**References**	**44**

Supplementary Table S1. Annotated list of the significantly associated variants with hypertension in the DISFIN study participants.

Index	Name	Chr	Position	GenTrain	MAF (%)	Coef	SD	T	P	Chi-Sq	P_raw	P_BH
578561	rs7200229	16	2956012	0.8207	36.21	430.98	6.37	67.64	4.11E-24	102.59	9.93E-15	1.10E-09
577534	rs7167587	15	37821357	0.8648	46.81	15.55	0.23	66.74	5.31E-24	102.09	1.15E-14	1.10E-09
489474	rs3098945	17	29597161	0.8531	18.54	22.60	0.34	66.72	5.34E-24	102.08	1.16E-14	1.10E-09
394845	rs1316826	18	48821785	0.8923	28.08	35.93	0.55	65.92	6.70E-24	101.63	1.32E-14	1.10E-09
417929	rs1514414	21	23686961	0.8599	37.10	17.39	0.27	65.45	7.69E-24	101.35	1.43E-14	1.10E-09
407372	rs1432019	8	128725872	0.9212	45.97	25.70	0.40	64.97	8.83E-24	101.08	1.55E-14	1.10E-09
12380	GSA-rs10496681	2	129925959	0.6018	35.27	16.96	0.27	63.71	1.28E-23	100.35	1.93E-14	1.10E-09
634359	rs916725	7	117280916	0.7889	49.62	11.78	0.19	63.67	1.30E-23	100.32	1.95E-14	1.10E-09
302760	GSA-rs9320772	6	120781189	0.9039	39.58	23.48	0.37	63.47	1.37E-23	100.21	2.02E-14	1.10E-09
565163	rs675828	13	102698383	0.8890	40.93	18.66	0.29	63.49	1.37E-23	100.22	2.01E-14	1.10E-09
636733	rs9314112	5	94459953	0.7956	47.38	15.97	0.25	62.84	1.66E-23	99.83	2.25E-14	1.10E-09
651707	seq-rs17258345	14	84757921	0.8850	40.06	24.35	0.39	62.68	1.74E-23	99.74	2.32E-14	1.10E-09
387508	rs12665877	7	78715145	0.8276	46.78	22.54	0.36	62.63	1.77E-23	99.71	2.34E-14	1.10E-09
240192	GSA-rs755228	10	75069616	0.8336	33.53	18.80	0.30	62.00	2.14E-23	99.33	2.61E-14	1.10E-09
476644	rs2665271	16	54093967	0.8490	46.61	18.25	0.29	61.86	2.23E-23	99.24	2.68E-14	1.10E-09
382648	rs12472254	2	34043547	0.8632	21.50	13.86	0.23	61.11	2.81E-23	98.79	3.07E-14	1.11E-09
300899	GSA-rs8183309	20	50460356	0.7163	44.77	16.13	0.26	61.07	2.85E-23	98.76	3.10E-14	1.11E-09
595015	rs7457005	7	82576862	0.8430	46.51	30.54	0.52	58.95	5.56E-23	97.44	4.59E-14	1.46E-09
333066	rs10075131	5	92712088	0.8522	47.75	34.35	0.58	58.74	5.93E-23	97.31	4.77E-14	1.46E-09
146808	GSA-rs2008026	8	96505859	0.9196	25.71	30.73	0.52	58.56	6.30E-23	97.19	4.94E-14	1.46E-09
517336	rs4681310	3	146264363	0.8669	27.19	21.81	0.37	58.26	6.93E-23	97.00	5.22E-14	1.46E-09
515658	rs4611977	4	187140313	0.8925	41.93	10.62	0.18	58.25	6.95E-23	96.99	5.23E-14	1.46E-09
357004	rs11247946	1	26392624	0.8262	42.47	17.10	0.30	57.77	8.13E-23	96.68	5.74E-14	1.50E-09
502223	rs3784729	15	92452777	0.7160	49.11	11.57	0.20	57.63	8.52E-23	96.59	5.90E-14	1.50E-09
373620	rs11996114	8	124417097	0.8032	46.76	-22.32	0.39	-57.00	1.05E-22	96.18	6.67E-14	1.55E-09
363105	rs11616092	12	8692897	0.8023	19.99	11.39	0.20	56.80	1.12E-22	96.05	6.93E-14	1.55E-09
562105	rs667335	10	118518065	0.7814	33.15	82.09	1.45	56.78	1.13E-22	96.03	6.96E-14	1.55E-09
424619	rs16932785	10	31925194	0.8558	38.49	40.26	0.71	56.69	1.16E-22	95.98	7.07E-14	1.55E-09
520480	rs4779012	15	79218567	0.8160	32.08	-21.37	0.38	-56.29	1.33E-22	95.71	7.66E-14	1.58E-09
471652	rs243147	14	62837918	0.8258	35.67	59.45	1.06	56.16	1.39E-22	95.63	7.85E-14	1.58E-09
509987	rs4282990	11	124516115	0.7937	38.18	37.41	0.67	56.06	1.43E-22	95.56	8.01E-14	1.58E-09
13119	GSA-rs1062246	3	11038483	0.8977	47.84	102.11	1.84	55.52	1.73E-22	95.19	8.93E-14	1.64E-09

419786	rs1560761	12	106083659	0.7744	43.61	18.56	0.34	55.40	1.80E-22	95.12	9.14E-14	1.64E-09
458627	rs2062541	16	16033378	0.8824	46.58	25.77	0.47	55.15	1.95E-22	94.95	9.61E-14	1.64E-09
613528	rs779746	3	7536844	0.8465	46.64	54.94	1.00	55.16	1.95E-22	94.95	9.59E-14	1.64E-09
374134	rs12025839	1	107983056	0.8133	25.31	30.05	0.55	54.98	2.07E-22	94.84	9.94E-14	1.64E-09
512322	rs4432101	12	55320315	0.8732	43.08	25.45	0.46	54.99	2.07E-22	94.84	9.93E-14	1.64E-09
554795	rs6432051	2	10028120	0.8822	33.20	158.64	2.91	54.57	2.39E-22	94.55	1.08E-13	1.74E-09
518556	rs4719137	7	71528863	0.7503	24.35	80.78	1.49	54.38	2.55E-22	94.42	1.12E-13	1.76E-09
96178	GSA-rs13182207	5	105759549	0.8867	45.72	100.00	1.85	54.13	2.78E-22	94.25	1.18E-13	1.78E-09
435178	rs17680472	13	70699512	0.9023	19.63	63.35	1.17	54.00	2.91E-22	94.16	1.21E-13	1.78E-09
395327	rs13196705	6	123439580	0.8537	41.51	15.06	0.28	53.71	3.22E-22	93.96	1.29E-13	1.78E-09
337873	rs10282010	7	53195861	0.8683	44.28	22.53	0.42	53.62	3.32E-22	93.90	1.31E-13	1.78E-09
418200	rs1522860	2	79050910	0.8149	12.99	19.29	0.36	53.46	3.51E-22	93.79	1.36E-13	1.78E-09
546018	rs608517	17	40579979	0.8212	33.77	27.14	0.51	53.44	3.55E-22	93.77	1.36E-13	1.78E-09
264836	GSA-rs77534060	7	48073440	0.9187	2.39	70.61	1.32	53.43	3.56E-22	93.76	1.37E-13	1.78E-09
523774	rs4866942	5	42641860	0.8303	38.27	38.94	0.73	53.42	3.57E-22	93.76	1.37E-13	1.78E-09
586009	rs73023553	6	169811409	0.8060	20.17	-9.13	0.17	-53.32	3.70E-22	93.68	1.40E-13	1.78E-09
474246	rs2544371	2	169370560	0.8206	45.71	31.30	0.59	53.18	3.89E-22	93.59	1.44E-13	1.80E-09
556811	rs6500936	16	7220581	0.8891	45.47	15.50	0.29	52.96	4.19E-22	93.44	1.51E-13	1.84E-09
302842	GSA-rs9328336	6	5919318	0.9191	39.55	33.90	0.65	52.06	5.81E-22	92.79	1.82E-13	2.10E-09
390937	rs1290005	12	94570381	0.7812	38.90	41.55	0.80	52.02	5.89E-22	92.76	1.84E-13	2.10E-09
389214	rs12767364	10	54913959	0.8227	22.66	-54.19	1.05	-51.82	6.35E-22	92.62	1.92E-13	2.10E-09
347165	rs10885198	10	111507867	0.9041	47.02	442.98	8.55	51.79	6.40E-22	92.60	1.93E-13	2.10E-09
629710	rs8091914	18	3192673	0.8079	41.25	10.08	0.19	51.79	6.40E-22	92.60	1.93E-13	2.10E-09
641202	rs9511108	13	24175505	0.8740	19.03	-41.36	0.80	-51.77	6.44E-22	92.59	1.94E-13	2.10E-09
476433	rs2654735	4	111477457	0.8752	34.06	33.59	0.65	51.73	6.56E-22	92.55	1.96E-13	2.10E-09
406276	rs1424005	16	64799112	0.8118	37.30	152.15	2.95	51.61	6.85E-22	92.47	2.01E-13	2.11E-09
467641	rs229527	22	37185445	0.8148	48.33	56.75	1.10	51.56	6.98E-22	92.43	2.03E-13	2.11E-09
550853	rs62105283	18	77377577	0.7796	14.08	-256.32	5.00	-51.30	7.66E-22	92.24	2.15E-13	2.17E-09
166802	GSA-rs3738706	1	115050199	0.8618	44.41	13.24	0.26	51.26	7.79E-22	92.21	2.17E-13	2.17E-09
582049	rs7267348	20	49514499	0.7480	29.37	76.21	1.49	51.16	8.06E-22	92.14	2.21E-13	2.18E-09
580819	rs7257166	19	17677995	0.7857	34.35	17.76	0.35	50.96	8.68E-22	92.00	2.31E-13	2.24E-09
607992	rs7697417	4	42213408	0.7334	31.76	23.03	0.45	50.73	9.47E-22	91.83	2.43E-13	2.26E-09
337786	rs10278730	7	138876514	0.8452	38.29	184.67	3.64	50.69	9.61E-22	91.80	2.45E-13	2.26E-09
104434	GSA-rs1412294	9	12926361	0.8848	45.68	41.12	0.81	50.68	9.66E-22	91.79	2.46E-13	2.26E-09
385348	rs12585338	13	22217020	0.8107	9.89	-209.28	4.13	-50.61	9.89E-22	91.74	2.49E-13	2.26E-09
271409	GSA-rs7806760	7	82718712	0.8754	43.80	58.89	1.16	50.58	1.00E-21	91.71	2.51E-13	2.26E-09
97721	GSA-rs13359266	5	25468702	0.8849	28.69	30.51	0.61	50.39	1.07E-21	91.57	2.62E-13	2.26E-09

520097	rs4768676	12	39239756	0.8747	37.64	28.90	0.57	50.34	1.09E-21	91.54	2.65E-13	2.26E-09
458012	rs2048265	4	124591556	0.8473	27.48	-20.81	0.41	-50.25	1.13E-21	91.47	2.70E-13	2.26E-09
520060	rs4767452	12	116779336	0.8060	20.63	-33.13	0.66	-50.23	1.14E-21	91.46	2.71E-13	2.26E-09
388513	rs12709705	18	40343378	0.7206	20.82	-186.32	3.71	-50.21	1.15E-21	91.44	2.73E-13	2.26E-09
490622	rs3132629	6	30378316	0.7978	44.32	21.83	0.43	50.21	1.15E-21	91.44	2.73E-13	2.26E-09
535363	rs58074807	7	10481712	0.7145	32.26	24.40	0.49	49.84	1.32E-21	91.17	2.95E-13	2.41E-09
384814	rs12564022	1	67205072	0.8174	41.72	252.87	5.09	49.72	1.38E-21	91.07	3.04E-13	2.43E-09
643887	rs9623671	22	42717428	0.8328	35.66	97.37	1.96	49.66	1.41E-21	91.03	3.08E-13	2.43E-09
514207	rs4546196	4	161032878	0.8608	18.49	29.86	0.60	49.64	1.43E-21	91.01	3.09E-13	2.43E-09
280467	GSA-rs7875641	9	112073549	0.8414	30.75	90.33	1.83	49.42	1.55E-21	90.85	3.25E-13	2.52E-09
457763	rs2041927	12	12527200	0.8770	47.83	-70.13	1.42	-49.27	1.64E-21	90.73	3.36E-13	2.57E-09
618419	rs7894962	10	128967528	0.8223	28.76	14.64	0.30	49.13	1.73E-21	90.63	3.47E-13	2.62E-09
90247	GSA-rs12468912	2	70640512	0.8871	42.16	44.87	0.91	49.05	1.78E-21	90.57	3.53E-13	2.63E-09
344896	rs10798510	1	177334492	0.8605	40.70	-56.13	1.15	-49.01	1.82E-21	90.54	3.56E-13	2.63E-09
479989	rs2796135	1	242392647	0.8727	20.63	13.51	0.28	48.95	1.86E-21	90.49	3.61E-13	2.63E-09
630677	rs8138412	22	44841212	0.7789	23.83	266.73	5.47	48.73	2.02E-21	90.32	3.80E-13	2.72E-09
275519	GSA-rs78406704	5	19446060	0.8107	5.88	329.31	6.76	48.70	2.05E-21	90.30	3.83E-13	2.72E-09
502234	rs378495	7	159159891	0.8688	44.61	85.29	1.76	48.59	2.13E-21	90.22	3.92E-13	2.76E-09
332456	rs1005060	15	91045317	0.8768	44.36	38.38	0.80	48.24	2.45E-21	89.94	4.25E-13	2.96E-09
551279	rs62191170	2	241888055	0.8442	17.84	-111.98	2.33	-48.05	2.64E-21	89.80	4.44E-13	3.05E-09
554293	rs638191	5	17183742	0.8073	14.18	-56.40	1.18	-47.82	2.89E-21	89.62	4.68E-13	3.19E-09
474371	rs2551043	8	3332374	0.8477	46.82	29.32	0.61	47.68	3.04E-21	89.52	4.83E-13	3.23E-09
601202	rs7579899	2	46310465	0.8640	49.21	45.60	0.96	47.66	3.07E-21	89.50	4.85E-13	3.23E-09
417531	rs1510795	4	90580463	0.7918	35.42	32.13	0.68	47.48	3.30E-21	89.36	5.06E-13	3.33E-09
14474	GSA-rs10816605	9	107992974	0.8627	32.86	25.30	0.53	47.38	3.44E-21	89.27	5.19E-13	3.35E-09
286298	GSA-rs79245693	2	134905275	0.8530	26.36	18.14	0.38	47.37	3.45E-21	89.27	5.20E-13	3.35E-09
551235	rs621846	1	9298184	0.8082	24.95	11.72	0.25	47.32	3.51E-21	89.23	5.25E-13	3.35E-09
412294	rs1470260	11	10253573	0.8396	21.27	13.82	0.29	47.17	3.74E-21	89.11	5.45E-13	3.44E-09
204887	GSA-rs717519	6	71641690	0.8940	47.91	-86.65	1.84	-47.08	3.88E-21	89.04	5.57E-13	3.48E-09
467330	rs229007	16	19204905	0.8231	43.87	22.78	0.48	46.97	4.04E-21	88.96	5.70E-13	3.53E-09
602655	rs7603307	2	115771679	0.8807	39.32	22.92	0.49	46.78	4.36E-21	88.80	5.97E-13	3.65E-09
463815	rs2223930	14	26031056	0.8115	44.14	20.57	0.44	46.64	4.61E-21	88.69	6.17E-13	3.74E-09
618540	rs7896909	10	131361291	0.7932	13.08	28.45	0.61	46.59	4.71E-21	88.65	6.25E-13	3.74E-09
467759	rs2297338	6	135054624	0.7504	48.87	63.96	1.37	46.56	4.77E-21	88.62	6.30E-13	3.74E-09
371028	rs11857443	15	100189501	0.7989	13.11	-123.94	2.68	-46.28	5.35E-21	88.40	6.73E-13	3.95E-09
366770	rs11718279	3	68673863	0.8592	47.47	12.93	0.28	46.21	5.49E-21	88.35	6.84E-13	3.95E-09
434384	rs17595186	4	99067562	0.8317	14.99	-42.74	0.93	-46.18	5.58E-21	88.32	6.90E-13	3.95E-09

570722	rs6939590	6	152929841	0.8076	25.80	55.48	1.20	46.17	5.59E-21	88.31	6.91E-13	3.95E-09
91963	GSA-rs12615167	2	48521908	0.8597	36.21	273.94	5.94	46.12	5.70E-21	88.27	6.99E-13	3.96E-09
472639	rs2480677	1	50111982	0.9112	10.74	-45.46	0.99	-46.09	5.78E-21	88.25	7.04E-13	3.96E-09
656493	seq-rs7178369	15	64743830	0.7893	34.26	-138.55	3.01	-45.98	6.04E-21	88.16	7.23E-13	4.02E-09
564396	rs6736756	2	172375419	0.9038	46.17	429.59	9.39	45.74	6.66E-21	87.97	7.66E-13	4.19E-09
376093	rs12141444	1	8329093	0.8802	6.67	-67.53	1.48	-45.74	6.67E-21	87.96	7.67E-13	4.19E-09
350363	rs11041816	11	8222251	0.7050	44.78	63.17	1.39	45.51	7.33E-21	87.78	8.10E-13	4.39E-09
355568	rs11204103	8	20238061	0.7906	41.38	32.40	0.71	45.41	7.63E-21	87.70	8.29E-13	4.42E-09
575981	rs7135160	12	22086364	0.8921	31.33	195.31	4.30	45.41	7.64E-21	87.69	8.30E-13	4.42E-09
169672	GSA-rs39504	8	89770681	0.8682	47.98	328.72	7.31	44.97	9.19E-21	87.33	9.25E-13	4.88E-09
389793	rs12818931	12	53678396	0.8436	6.71	-25.85	0.58	-44.83	9.75E-21	87.21	9.58E-13	5.01E-09
54805	GSA-rs1170192	13	42099217	0.9004	30.24	37.25	0.83	44.63	1.06E-20	87.05	1.01E-12	5.19E-09
598791	rs7538519	1	195417767	0.8784	27.67	28.29	0.63	44.62	1.06E-20	87.04	1.01E-12	5.19E-09
510087	rs4289215	2	163167589	0.8337	48.46	-212.64	4.78	-44.52	1.11E-20	86.96	1.03E-12	5.28E-09
555053	rs6441034	3	156138408	0.7781	42.34	26.24	0.59	44.38	1.18E-20	86.84	1.07E-12	5.42E-09
381852	rs12443114	15	97250458	0.8136	39.14	221.42	5.00	44.30	1.22E-20	86.77	1.09E-12	5.48E-09
18704	GSA-rs11128922	3	19810896	0.7910	25.45	44.73	1.01	44.25	1.25E-20	86.73	1.11E-12	5.48E-09
558259	rs6547930	2	29450074	0.8628	46.38	142.36	3.22	44.23	1.25E-20	86.72	1.11E-12	5.48E-09
470670	rs2398442	12	128978365	0.8398	37.07	-121.30	2.75	-44.03	1.37E-20	86.55	1.17E-12	5.72E-09
642761	rs9566255	13	37804227	0.8328	23.85	48.20	1.10	43.97	1.40E-20	86.49	1.19E-12	5.76E-09
86162	GSA-rs12032340	1	171766058	0.9097	21.94	66.50	1.52	43.64	1.61E-20	86.22	1.29E-12	6.21E-09
387659	rs12670839	7	148261752	0.8285	42.28	44.69	1.03	43.50	1.72E-20	86.09	1.34E-12	6.40E-09
38059	GSA-rs115345471	1	37673773	0.8278	11.72	-34.12	0.79	-43.41	1.78E-20	86.02	1.37E-12	6.49E-09
395894	rs13236240	7	7051994	0.8150	47.75	32.68	0.75	43.36	1.82E-20	85.98	1.38E-12	6.52E-09
382729	rs12475645	2	132996787	0.8542	35.06	68.64	1.59	43.14	2.01E-20	85.78	1.47E-12	6.85E-09
542805	rs59870410	19	22301161	0.5508	23.03	319.63	7.44	42.98	2.15E-20	85.65	1.53E-12	7.07E-09
166876	GSA-rs3740057	10	99898828	0.8755	41.53	43.45	1.01	42.92	2.21E-20	85.59	1.55E-12	7.14E-09
85408	GSA-rs11942693	4	165009016	0.8806	27.67	-25.20	0.59	-42.76	2.37E-20	85.45	1.62E-12	7.38E-09
643675	rs960995	19	56527800	0.8786	47.41	40.25	0.95	42.58	2.57E-20	85.30	1.70E-12	7.69E-09
12345	GSA-rs10494876	1	206436768	0.8158	9.83	-383.04	9.02	-42.45	2.72E-20	85.19	1.75E-12	7.88E-09
379396	rs12275946	11	11310176	0.8246	43.88	-192.90	4.56	-42.33	2.87E-20	85.08	1.81E-12	8.08E-09
335004	rs10160607	11	21391411	0.8572	37.77	66.90	1.58	42.21	3.02E-20	84.98	1.86E-12	8.26E-09
528669	rs530271	20	10086616	0.7884	47.76	53.80	1.28	42.08	3.21E-20	84.86	1.93E-12	8.50E-09
170360	GSA-rs41276672	12	32582859	0.9205	9.36	94.78	2.25	42.05	3.24E-20	84.83	1.94E-12	8.50E-09
417397	rs1509731	11	109134639	0.8371	9.71	-69.24	1.65	-41.89	3.48E-20	84.69	2.03E-12	8.80E-09
332287	rs10044159	5	59118221	0.8916	17.35	65.14	1.56	41.76	3.70E-20	84.58	2.10E-12	9.05E-09
521375	rs4801933	19	38894658	0.7903	48.24	95.60	2.30	41.66	3.88E-20	84.48	2.16E-12	9.24E-09

278797	GSA-rs7864748	9	89098472	0.8175	22.88	96.95	2.36	41.11	4.96E-20	84.00	2.49E-12	1.06E-08
350694	rs1105586	13	43658497	0.8908	25.16	882.53	21.72	40.63	6.21E-20	83.55	2.85E-12	1.20E-08
177630	GSA-rs4948446	10	61244747	0.8628	36.73	348.11	8.62	40.40	6.88E-20	83.35	3.03E-12	1.26E-08
361040	rs115401064	6	33021561	0.8322	9.76	80.53	1.99	40.39	6.91E-20	83.34	3.03E-12	1.26E-08
362051	rs11582753	1	203014166	0.8035	44.30	-54.30	1.35	-40.18	7.63E-20	83.14	3.22E-12	1.33E-08
177571	GSA-rs4942380	13	44924336	0.9129	35.68	197.33	4.93	40.04	8.14E-20	83.01	3.34E-12	1.37E-08
367334	rs11734394	4	76719464	0.8032	47.96	58.09	1.47	39.50	1.05E-19	82.51	3.88E-12	1.58E-08
598801	rs7538861	1	48734735	0.8403	40.18	255.70	6.52	39.23	1.20E-19	82.25	4.19E-12	1.70E-08
205417	GSA-rs7227018	18	34996129	0.8760	25.00	74.86	1.93	38.79	1.48E-19	81.84	4.75E-12	1.91E-08
628622	rs8056747	16	55872575	0.8100	49.76	57.40	1.49	38.47	1.73E-19	81.53	5.20E-12	2.08E-08
504029	rs3828893	6	31480876	0.8038	4.86	-680.75	17.97	-37.89	2.30E-19	80.96	6.16E-12	2.45E-08
563479	rs671290	11	78956891	0.9037	45.83	51.79	1.40	37.10	3.40E-19	80.19	7.76E-12	3.06E-08
293712	GSA-rs79848991	19	55935055	0.8195	2.60	113.94	3.09	36.85	3.87E-19	79.93	8.37E-12	3.28E-08
383915	rs12527715	6	32849221	0.7614	29.86	63.82	1.74	36.61	4.38E-19	79.69	9.00E-12	3.51E-08
587681	rs7317149	13	76630179	0.8182	21.28	-123.95	3.43	-36.16	5.52E-19	79.23	1.03E-11	3.99E-08
33578	GSA-rs1147199	9	84660980	0.8537	31.73	65.61	1.82	36.09	5.74E-19	79.16	1.05E-11	4.06E-08
15034	GSA-rs10865196	2	43962491	0.8964	48.25	-703.99	19.63	-35.86	6.44E-19	78.93	1.13E-11	4.32E-08
578401	rs7194812	16	81279361	0.8627	28.44	261.09	7.31	35.72	6.95E-19	78.78	1.18E-11	4.49E-08
575470	rs7120515	11	117121210	0.8603	35.11	42.84	1.27	33.83	1.92E-18	76.77	2.15E-11	8.11E-08
91789	GSA-rs12599402	16	11096031	0.8704	41.72	583.23	17.62	33.10	2.89E-18	75.97	2.73E-11	1.02E-07
555617	rs6459707	7	155512409	0.8088	42.60	30.11	0.91	33.09	2.90E-18	75.96	2.74E-11	1.02E-07
473667	rs2522288	22	17467130	0.8664	21.27	1.76E+14	5.42E+12	32.39	4.33E-18	75.16	3.47E-11	1.29E-07
509951	rs4281012	7	67394986	0.8830	22.86	39.29	1.22	32.21	4.81E-18	74.96	3.69E-11	1.36E-07
419206	rs154802	5	92741424	0.8007	26.83	50.98	1.63	31.36	7.91E-18	73.98	4.95E-11	1.81E-07
2202	chr1:74673341	1	74207657	0.7965	44.31	8.79E+13	3.36E+12	26.18	2.27E-16	67.35	3.58E-10	1.30E-06
399167	rs1353663	4	81738246	0.8718	30.37	-3.51E+14	1.53E+13	-22.98	2.52E-15	62.61	1.48E-09	5.36E-06
302956	GSA-rs9346239	6	68764602	0.8899	37.15	119.72	7.43	16.11	1.56E-12	49.97	6.60E-08	2.38E-04
78015	GSA-rs11793324	9	93951408	0.7885	12.47	3.26E+14	2.11E+13	15.44	3.29E-12	48.51	1.02E-07	3.67E-04
104148	GSA-rs141071850	5	79380040	0.6971	1.54	291.93	19.34	15.09	4.95E-12	47.71	1.30E-07	4.64E-04
618418	rs7894961	10	5314899	0.8140	42.73	2.34E+14	1.59E+13	14.68	8.07E-12	46.75	1.74E-07	6.17E-04
14956	GSA-rs10858101	9	135424709	0.7418	40.81	2.67E+14	1.86E+13	14.38	1.16E-11	46.05	2.16E-07	7.58E-04
120750	GSA-rs150809754	1	231291000	0.5484	8.39	0.41	0.03	13.67	2.78E-11	44.32	3.63E-07	1.27E-03
418161	rs1521776	8	105029599	0.9107	46.49	1.66E+14	1.31E+13	12.69	1.01E-10	41.81	7.76E-07	2.70E-03
386669	rs12637115	3	3645905	0.8223	34.64	4.51E+14	3.76E+13	12.00	2.59E-10	39.96	1.36E-06	4.71E-03
531802	rs56207641	16	30751184	0.6511	0.84	0.46	0.04	11.45	5.73E-10	38.41	2.18E-06	7.49E-03
137279	GSA-rs182593061	5	119428913	0.5154	5.35	0.65	0.06	10.85	1.39E-09	36.69	3.68E-06	1.26E-02

314298	rs371712630.1	12	51663087	0.5547	0.79	-3.50	0.33	-10.73	1.68E-09	36.31	4.13E-06	1.40E-02
544434	rs6035868	20	21395188	0.8472	35.82	2.63E+14	2.46E+13	10.66	1.87E-09	36.10	4.40E-06	1.49E-02
652420	seq-rs28933396	19	38499997	0.6202	1.74	-4.20	0.40	-10.39	2.82E-09	35.31	5.61E-06	1.89E-02
416897	rs150593977	6	135586046	0.7674	1.77	4.06	0.39	10.35	3.03E-09	35.16	5.86E-06	1.96E-02
264463	GSA-rs77505745	13	52012786	0.8929	12.69	1.26E+14	1.23E+13	10.24	3.58E-09	34.84	6.47E-06	2.14E-02
595702	rs74740987	22	50414592	0.7283	2.11	3.56	0.35	10.24	3.58E-09	34.84	6.47E-06	2.14E-02
271888	GSA-rs78107974	1	76043626	0.6010	1.47	17.66	1.73	10.22	3.68E-09	34.79	6.57E-06	2.16E-02
71272	GSA-rs117667022	1	70828940	0.8234	1.75	57.98	5.69	10.20	3.85E-09	34.70	6.75E-06	2.21E-02
144776	GSA-rs192052273	10	67797769	0.5544	0.78	28.59	2.85	10.02	5.08E-09	34.16	7.96E-06	2.59E-02
265336	GSA-rs77574509	1	213432612	0.7478	2.39	2.68	0.27	10.01	5.21E-09	34.11	8.09E-06	2.62E-02
109005	GSA-rs143900396	22	50205242	0.7352	2.79	1.16	0.12	9.97	5.52E-09	34.00	8.36E-06	2.69E-02
664774	19:41526116_CYP2B6	19	41020211	0.0000	9.06	0.50	0.05	9.87	6.54E-09	33.67	9.25E-06	2.96E-02

Index, index of the variant in the GSA; Chr, chromosome; MAF, minor allele frequency; Coef, regression coefficient from the random effects logistic regression; SD, standard deviation; P_raw, p value unadjusted for multiple comparisons; P_BH, false discovery rate adjusted p value using the Benjamini-Hochberg procedure

Supplementary Table S2. Annotation of the 149 named genes related to the 191 significantly associated variants shown in Supplementary Table S1.

No	Gene	Annotation
1	*ANKRD13B*	Ankyrin repeat domain-containing protein 13B; Ubiquitin-binding protein that specifically recognizes and binds 'Lys-63'-linked ubiquitin. Does not bind 'Lys-48'-linked ubiquitin. Positively regulates the internalization of ligand-activated EGFR by binding to the Ub moiety of ubiquitinated EGFR at the cell membrane.
2	*MAGI2*	Membrane-associated guanylate kinase, WW and PDZ domain-containing protein 2; Seems to act as scaffold molecule at synaptic junctions by assembling neurotransmitter receptors and cell adhesion proteins. May play a role in regulating activin-mediated signaling in neuronal cells. Enhances the ability of PTEN to suppress AKT1 activation. Plays a role in nerve growth factor (NGF)-induced recruitment of RAPGEF2 to late endosomes and neurite outgrowth.
3	*DUPD1*	Dual specificity phosphatase DUPD1; Dual specificity phosphatase able to dephosphorylate phosphotyrosine, phosphoserine and phosphothreonine residues, with a preference for phosphotyrosine as a substrate. Belongs to the protein-tyrosine phosphatase family. Non- receptor class dual specificity subfamily.
4	*FTO*	Alpha-ketoglutarate-dependent dioxygenase FTO; RNA demethylase that mediates oxidative demethylation of different RNA species, such as mRNAs, tRNAs and snRNAs, and acts as a regulator of fat mass, adipogenesis and energy homeostasis. Specifically demethylates N(6)- methyladenosine (m6A) RNA, the most prevalent internal modification of messenger RNA (mRNA) in higher eukaryotes. M6A demethylation by FTO affects mRNA expression and stability. Also able to demethylate m6A in U6 small nuclear RNA (snRNA). Mediates demethylation of N(6),2'-O- dimethyladenosine cap (m6A(m)), by demethylating [...]
5	*SDC2*	Syndecan-2; Cell surface proteoglycan that bears heparan sulfate. Regulates dendritic arbor morphogenesis (By similarity).
6	*PLSCR4*	Phospholipid scramblase 4; May mediate accelerated ATP-independent bidirectional transbilayer migration of phospholipids upon binding calcium ions that results in a loss of phospholipid asymmetry in the plasma membrane. May play a central role in the initiation of fibrin clot formation, in the activation of mast cells and in the recognition of apoptotic and injured cells by the reticuloendothelial system.

7	*ZNF683*	Tissue-resident T-cell transcription regulator protein ZNF683; Transcription factor that mediates a transcriptional program in various innate and adaptive immune tissue-resident lymphocyte T-cell types such as tissue-resident memory T (Trm), natural killer (trNK) and natural killer T (NKT) cells and negatively regulates gene expression of proteins that promote the egress of tissue-resident T-cell populations from non-lymphoid organs. Plays a role in the development, retention and long-term establishment of adaptive and innate tissue- resident lymphocyte T cell types in non-lymphoid org [...]
8	*LIN28A*	Protein lin-28 homolog A; RNA-binding protein that inhibits processing of pre-let-7 miRNAs and regulates translation of mRNAs that control developmental timing, pluripotency and metabolism. Seems to recognize a common structural G-quartet (G4) feature in its miRNA and mRNA targets (Probable). 'Translational enhancer' that drives specific mRNAs to polysomes and increases the efficiency of protein synthesis. Its association with the translational machinery and target mRNAs results in an increased number of initiation events per molecule of mRNA and, indirectly, in mRNA stabilization. Bin [...]
9	*ST8SIA2*	Alpha-2,8-sialyltransferase 8B; May transfer sialic acid through alpha-2,8-linkages to the alpha-2,3-linked and alpha-2,6-linked sialic acid of N-linked oligosaccharides of glycoproteins and may be involved in PSA (polysialic acid) expression; Belongs to the glycosyltransferase 29 family.
10	*TRMT12*	tRNA wybutosine-synthesizing protein 2 homolog; S-adenosyl-L-methionine-dependent transferase that acts as a component of the wybutosine biosynthesis pathway. Wybutosine is a hyper modified guanosine with a tricyclic base found at the 3'-position adjacent to the anticodon of eukaryotic phenylalanine tRNA. Catalyzes the transfer of the alpha-amino-alpha-carboxypropyl (acp) group from S- adenosyl-L-methionine to the C-7 position of 4-demethylwyosine (imG-14) to produce wybutosine-86; Belongs to the class I-like SAM-binding methyltransferase superfamily. TRM5/TYW2 family.
11	*TMEM65*	Transmembrane protein 65; May play an important role in cardiac development and function. May regulate cardiac conduction and the function of the gap junction protein GJA1. May contribute to the stability and proper localization of GJA1 to cardiac intercalated disk thereby regulating gap junction communication (By similarity). May also play a role in the regulation of mitochondrial respiration and mitochondrial DNA copy number maintenance.
12	*RIMKLB*	Beta-citrylglutamate synthase B; Catalyzes the synthesis of beta-citryl-L-glutamate and N- acetyl-L-aspartyl-L-glutamate. Beta-citryl-L-glutamate is synthesized more efficiently than N-acetyl-L-aspartyl-L-glutamate. Belongs to the RimK family.
13	*ARHGAP12*	Rho GTPase-activating protein 12; GTPase activator for the Rho-type GTPases by converting them to an inactive GDP-bound state.
14	*KCNH5*	Potassium voltage-gated channel subfamily H member 5; Pore-forming (alpha) subunit of voltage-gated potassium channel. Elicits a non-inactivating outward rectifying current. Channel properties may be

		modulated by cAMP and subunit assembly; Belongs to the potassium channel family. H (Eag) (TC 1.A.1.20) subfamily. Kv10.2/KCNH5 sub-subfamily.
15	*OR8B8*	Olfactory receptor 8B8; Odorant receptor (Potential). May be involved in taste perception.
16	*SLC6A1*	Sodium- and chloride-dependent GABA transporter 1; Terminates the action of GABA by its high affinity sodium- dependent reuptake into presynaptic terminals.
17	*NUAK1*	NUAK family SNF1-like kinase 1; Serine/threonine-protein kinase involved in various processes such as cell adhesion, regulation of cell ploidy and senescence, cell proliferation and tumor progression. Phosphorylates ATM, CASP6, LATS1, PPP1R12A and p53/TP53. Acts as a regulator of cellular senescence and cellular ploidy by mediating phosphorylation of 'Ser-464' of LATS1, thereby controlling its stability. Controls cell adhesion by regulating activity of the myosin protein phosphatase 1 (PP1) complex. Acts by mediating phosphorylation of PPP1R12A subunit of myosin PP1: phosphorylated PPP [...]
18	*GRM7*	Metabotropic glutamate receptor 7; G-protein coupled receptor for glutamate. Ligand binding causes a conformation change that triggers signaling via guanine nucleotide-binding proteins (G proteins) and modulates the activity of down-stream effectors, such as adenylate cyclase. Signaling inhibits adenylate cyclase activity.
19	*ABCC1*	Multidrug resistance-associated protein 1; Mediates export of organic anions and drugs from the cytoplasm. Mediates ATP-dependent transport of glutathione and glutathione conjugates, leukotriene C4, estradiol-17-beta-o-glucuronide, methotrexate, antiviral drugs and other xenobiotics. Confers resistance to anticancer drugs by decreasing accumulation of drug in cells, and by mediating ATP- and GSH-dependent drug export. Hydrolyzes ATP with low efficiency. Catalyzes the export of sphingosine 1-phosphate from mast cells independently of their degranulation. Participates in inflammatory re [...]
20	*OR6C1*	Olfactory receptor 6C1; Odorant receptor; Belongs to the G-protein coupled receptor 1 family.
21	*GALNT17*	Polypeptide N-acetylgalactosaminyltransferase 17; May catalyze the initial reaction in O-linked oligosaccharide biosynthesis, the transfer of an N-acetyl-D-galactosamine residue to a serine or threonine residue on the protein receptor. Belongs to the glycosyltransferase 2 family. GalNAc-T subfamily.
22	*TRDN*	Triadin; Contributes to the regulation of lumenal Ca2+ release via the sarcoplasmic reticulum calcium release channels RYR1 and RYR2, a key step in triggering skeletal and heart muscle contraction. Required for normal organization of the triad junction, where T-tubules and the sarcoplasmic reticulum terminal cisternae are in close contact (By similarity). Required for normal skeletal muscle strength. Plays a role in excitation-contraction coupling in the heart and in regulating the rate of heart beats.

23	*REG3G*	Regenerating islet-derived protein 3-gamma 16.5 kDa form; Bactericidal C-type lectin which acts exclusively against Gram-positive bacteria and mediates bacterial killing by binding to surface-exposed carbohydrate moieties of peptidoglycan. Restricts bacterial colonization of the intestinal epithelial surface and consequently limits activation of adaptive immune responses by the microbiota. The uncleaved form has bacteriostatic activity, whereas the cleaved form has bactericidal activity against L.monocytogenes and methicillin-resistant S.aureus. Regulates keratinocyte proliferation and [...]
24	*REG1B*	Lithostathine-1-beta; Might act as an inhibitor of spontaneous calcium carbonate precipitation. May be associated with neuronal sprouting in brain, and with brain and pancreas regeneration.
25	*CCR7*	C-C chemokine receptor type 7; Receptor for the MIP-3-beta chemokine. Probable mediator of EBV effects on B-lymphocytes or of normal lymphocyte functions; Belongs to the G-protein coupled receptor 1 family.
26	*C7orf57*	Uncharacterized protein C7orf57; Chromosome 7 open reading frame 57.
27	*GHR*	Growth hormone-binding protein; Receptor for pituitary gland growth hormone involved in regulating postnatal body growth. On ligand binding, couples to the JAK2/STAT5 pathway (By similarity). Isoform 2 up-regulates the production of GHBP and acts as a negative inhibitor of GH signaling.
28	*CCDC152*	Coiled-coil domain containing 152.
29	*SELENOP*	Selenoprotein P; Might be responsible for some of the extracellular antioxidant defense properties of selenium or might be involved in the transport of selenium. May supply selenium to tissues such as brain and testis.
30	*LRP2*	Low-density lipoprotein receptor-related protein 2; Multiligand endocytic receptor (By similarity). Acts together with CUBN to mediate endocytosis of high-density lipoproteins (By similarity). Mediates receptor-mediated uptake of polybasic drugs such as aprotinin, aminoglycosides and polymyxin B (By similarity). In the kidney, mediates the tubular uptake and clearance of leptin (By similarity). Also mediates transport of leptin across the blood-brain barrier through endocytosis at the choroid plexus epithelium (By similarity). Endocytosis of leptin in neuronal cells is required for hyp [...]
31	*RBFOX1*	RNA binding protein fox-1 homolog 1; RNA-binding protein that regulates alternative splicing events by binding to 5'-UGCAUGU-3' elements. Regulates alternative splicing of tissue-specific exons and of differentially spliced exons during erythropoiesis.
32	*TMCC3*	Transmembrane and coiled-coil domain family 3; Belongs to the TEX28 family.
33	*PCDH15*	Protocadherin-15; Calcium-dependent cell-adhesion protein. Essential for maintenance of normal retinal and cochlear function.
34	*MYOM1*	Myomesin-1; Major component of the vertebrate myofibrillar M band. Binds myosin, titin, and light meromyosin. This binding is dose dependent.

35	*SPATA13*	Spermatogenesis-associated protein 13; Acts as guanine nucleotide exchange factor (GEF) for RHOA, RAC1 and CDC42 GTPases. Regulates cell migration and adhesion assembly and disassembly through a RAC1, PI3K, RHOA and AKT1-dependent mechanism. Increases both RAC1 and CDC42 activity, but decreases the amount of active RHOA. Required for MMP9 up-regulation via the JNK signaling pathway in colorectal tumor cells. Involved in tumor angiogenesis and may play a role in intestinal adenoma formation and tumor progression.
36	*C1QTNF6*	Complement C1q tumor necrosis factor-related protein 6; C1q and TNF related 6.
37	*TSPAN2*	Tetraspanin-2; May play a role in signalling in oligodendrocytes in the early stages of their terminal differentiation into myelin-forming glia and may also function in stabilizing the mature sheath; Belongs to the tetraspanin (TM4SF) family.
38	*TSHB*	Thyrotropin subunit beta; Indispensable for the control of thyroid structure and metabolism; Belongs to the glycoprotein hormones subunit beta family.
39	*PTGIS*	Prostacyclin synthase; Catalyzes the isomerization of prostaglandin H2 to prostacyclin (= prostaglandin I2).
40	*UNC13A*	Protein unc-13 homolog A; Plays a role in vesicle maturation during exocytosis as a target of the diacylglycerol second messenger pathway. Involved in neurotransmitter release by acting in synaptic vesicle priming prior to vesicle fusion and participates in the activity-dependent refilling of readily releasable vesicle pool (RRP). Essential for synaptic vesicle maturation in most excitatory/glutamatergic but not inhibitory/GABA- mediated synapses. Facilitates neuronal dense core vesicles fusion as well as controls the location and efficiency of their synaptic release (By similarity). A [...]
41	*KIAA1549*	UPF0606 protein KIAA1549; KIAA1549; Belongs to the UPF0606 family.
42	*PCLO*	Protein piccolo; Scaffold protein of the presynaptic cytomatrix at the active zone (CAZ) which is the place in the synapse where neurotransmitter is released (By similarity). After synthesis, participates in the formation of Golgi-derived membranous organelles termed Piccolo-Bassoon transport vesicles (PTVs) that are transported along axons to sites of nascent synaptic contacts (By similarity). At the presynaptic active zone, regulates the spatial organization of synaptic vesicle cluster, the protein complexes that execute membrane fusion and compensatory endocytosis (By similarity). O [...]
43	*KIF21A*	Kinesin-like protein KIF21A; Microtubule-binding motor protein probably involved in neuronal axonal transport. In vitro, has a plus-end directed motor activity (By similarity).
44	*RNFT2*	Ring finger protein, transmembrane 2.
45	*IL23R*	Interleukin-23 receptor; Associates with IL12RB1 to form the interleukin-23 receptor. Binds IL23 and mediates T-cells, NK cells and possibly certain macrophage/myeloid cells stimulation probably through activation of the Jak-Stat signaling cascade. IL23 functions in innate and adaptive immunity and may participate in acute response to infection in peripheral tissues. IL23 may be responsible for autoimmune

		inflammatory diseases and be important for tumorigenesis. Belongs to the type I cytokine receptor family. Type 2 subfamily.
46	*C1orf141*	Uncharacterized protein C1orf141; Chromosome 1 open reading frame 141.
47	*A4GALT*	Lactosylceramide 4-alpha-galactosyltransferase; Catalyzes the transfer of galactose from UDP-alpha-D-galactose to lactosylceramide/beta-D-galactosyl-(1->4)-beta-D-glucosyl- (1<->1)-ceramide(d18:1(4E)) to produce globotriaosylceramide/globoside Gb3Cer (d18:1(4E)). Also able to transfer galactose to galactosylceramide/beta-D-Gal-(1<->1')-Cer. Globoside Gb3Cer is a glycosphingolipid of the globo serie, one of the major types of neutral root structures of glycosphingolipids, that constitute a significant portion of mammalian cell membranes (Probable). Globotriaosylceramide/globoside Gb3Ce [...]
48	*CYB5R3*	NADH-cytochrome b5 reductase 3 membrane-bound form; Desaturation and elongation of fatty acids, cholesterol biosynthesis, drug metabolism, and, in erythrocyte, methemoglobin reduction.
49	*SUSD1*	Sushi domain containing 1.
50	*DUSP16*	Dual specificity protein phosphatase 16; Dual specificity protein phosphatase involved in the inactivation of MAP kinases. Dephosphorylates MAPK10 bound to ARRB2.
51	*ADD2*	Beta-adducin; Membrane-cytoskeleton-associated protein that promotes the assembly of the spectrin-actin network. Binds to the erythrocyte membrane receptor SLC2A1/GLUT1 and may therefore provide a link between the spectrin cytoskeleton to the plasma membrane. Binds to calmodulin. Calmodulin binds preferentially to the beta subunit. Belongs to the aldolase class II family. Adducin subfamily.
52	*PLD5*	Inactive phospholipase D5; Phospholipase D family member 5; Belongs to the phospholipase D family.
53	*ARHGAP8*	Rho GTPase-activating protein 8; GTPase activator for the Rho-type GTPases by converting them to an inactive GDP-bound state.
54	*CDH18*	Cadherin-18; Cadherins are calcium-dependent cell adhesion proteins. They preferentially interact with themselves in a homophilic manner in connecting cells; cadherins may thus contribute to the sorting of heterogeneous cell types.
55	*VIPR2*	Vasoactive intestinal polypeptide receptor 2; This is a receptor for VIP as well as PACAP-38 and -27, the activity of this receptor is mediated by G proteins which activate adenylyl cyclase. Can be coupled to phospholipase C.
56	*RTP5*	Receptor transporter protein 5.
57	*FAM240C*	Protein FAM240C; Family with sequence similarity 240 member C; Belongs to the FAM240 family.

58	*BASP1*	Brain abundant membrane attached signal protein 1; Belongs to the BASP1 family.
59	*CSMD1*	CUB and sushi domain-containing protein 1; Potential suppressor of squamous cell carcinomas.
60	*EPAS1*	Endothelial PAS domain-containing protein 1; Transcription factor involved in the induction of oxygen regulated genes. Heterodimerizes with ARNT; heterodimer binds to core DNA sequence 5'-TACGTG-3' within the hypoxia response element (HRE) of target gene promoters (By similarity). Regulates the vascular endothelial growth factor (VEGF) expression and seems to be implicated in the development of blood vessels and the tubular system of lung. May also play a role in the formation of the endothelium that gives rise to the blood brain barrier. Potent activator of the Tie-2 tyrosine kinase e [...]
61	*CCSER1*	Coiled-coil serine rich protein 1.
62	*CCNT2*	Cyclin-T2; Regulatory subunit of the cyclin-dependent kinase pair (CDK9/cyclin T) complex, also called positive transcription elongation factor B (P-TEFB), which is proposed to facilitate the transition from abortive to production elongation by phosphorylating the CTD (carboxy- terminal domain) of the large subunit of RNA polymerase II (RNAP II). The activity of this complex is regulated by binding with 7SK snRNA. Plays a role during muscle differentiation; P-TEFB complex interacts with MYOD1; this tripartite complex promotes the transcriptional activity of MYOD1 through its CDK9-media [...]
63	*ACMSD*	2-amino-3-carboxymuconate-6-semialdehyde decarboxylase; Converts alpha-amino-beta-carboxymuconate-epsilon- semialdehyde (ACMS) to alpha-aminomuconate semialdehyde (AMS). ACMS can be converted non-enzymatically to quinolate (QA), a key precursor of NAD, and a potent endogenous excitotoxin of neuronal cells which is implicated in the pathogenesis of various neurodegenerative disorders. In the presence of ACMSD, ACMS is converted to AMS, a benign catabolite. ACMSD ultimately controls the metabolic fate of tryptophan catabolism along the kynurenine pathway; Belongs to the metallo-dependent [...]
64	*MAP3K19*	Mitogen-activated protein kinase kinase kinase 19.
65	*SPSB1*	SPRY domain-containing SOCS box protein 1; Substrate recognition component of a SCF-like ECS (Elongin BC-CUL2/5-SOCS-box protein) E3 ubiquitin-protein ligase complex which mediates the ubiquitination and subsequent proteasomal degradation of target proteins. Negatively regulates nitric oxide (NO) production and limits cellular toxicity in activated macrophages by mediating the ubiquitination and proteasomal degradation of NOS2. Acts as a bridge which links NOS2 with the ECS E3 ubiquitin ligase complex components ELOC and CUL5.

66	*SBF2*	Myotubularin-related protein 13; Guanine nucleotide exchange factor (GEF) which activates RAB21 and possibly RAB28. Promotes the exchange of GDP to GTP, converting inactive GDP-bound Rab proteins into their active GTP-bound form. In response to starvation-induced autophagy, activates RAB21 which in turn binds to and regulates SNARE protein VAMP8 endolysosomal transport required for SNARE-mediated autophagosome-lysosome fusion. Acts as an adapter for the phosphatase MTMR2 (By similarity). Increases MTMR2 catalytic activity towards phosphatidylinositol 3,5-bisphosphate and to a lesser ex [...]
67	*SYT17*	Synaptotagmin-17; Plays a role in dendrite formation by melanocytes ; Belongs to the synaptotagmin family.
68	*DPP10*	Inactive dipeptidyl peptidase 10; Promotes cell surface expression of the potassium channel KCND2. Modulates the activity and gating characteristics of the potassium channel KCND2. Has no dipeptidyl aminopeptidase activity.
69	*TCERG1L*	Transcription elongation regulator 1 like.
70	*HBS1L*	HBS1-like protein; HBS1 like translational GTPase; Belongs to the TRAFAC class translation factor GTPase superfamily. Classic translation factor GTPase family.
71	*ADAMTS17*	ADAM metallopeptidase with thrombospondin type 1 motif 17.
72	*PPP1R21*	Protein phosphatase 1 regulatory subunit 21; Putative regulator of protein phosphatase 1 (PP1) activity. May play a role in the endosomal sorting process or in endosome maturation pathway.
73	*ELAVL4*	ELAV-like protein 4; RNA-binding protein that is involved in the post- transcriptional regulation of mRNAs. Plays a role in the regulation of mRNA stability, alternative splicing and translation. Binds to AU-rich element (ARE) sequences in the 3' untranslated region (UTR) of target mRNAs, including GAP43, VEGF, FOS, CDKN1A and ACHE mRNA. Many of the target mRNAs are coding for RNA-binding proteins, transcription factors and proteins involved in RNA processing and/or neuronal development and function (By similarity). By binding to the mRNA 3'UTR, decreases mRNA deadenylation and thereby [...]
74	*RBPMS2*	RNA-binding protein with multiple splicing 2; RNA-binding protein involved in the regulation of smooth muscle cell differentiation and proliferation in the gastrointestinal system. Binds NOG mRNA, the major inhibitor of the bone morphogenetic protein (BMP) pathway. Mediates an increase of NOG mRNA levels, thereby contributing to the negative regulation of BMP signaling pathway and promoting reversible dedifferentiation and proliferation of smooth muscle cells (By similarity).
75	*ZNF609*	Zinc finger protein 609; Transcription factor, which activates RAG1, and possibly RAG2, transcription. Through the regulation of RAG1/2 expression, may regulate thymocyte maturation. Along with NIPBL and the multiprotein complex Integrator, promotes cortical neuron migration during brain development by regulating the transcription of crucial genes in this process. Preferentially binds promoters containing paused RNA polymerase II. Up-regulates the expression of SEMA3A, NRP1, PLXND1 and GABBR2 genes, among others.

76	*PIF1*	ATP-dependent DNA helicase PIF1; DNA-dependent ATPase and 5'-3' DNA helicase required for the maintenance of both mitochondrial and nuclear genome stability. Efficiently unwinds G-quadruplex (G4) DNA structures and forked RNA-DNA hybrids. Resolves G4 structures, preventing replication pausing and double-strand breaks (DSBs) at G4 motifs. Involved in the maintenance of telomeric DNA. Inhibits telomere elongation, de novo telomere formation and telomere addition to DSBs via catalytic inhibition of telomerase. Reduces the processivity of telomerase by displacing active telomerase from DNA [...]
77	*ITGA6*	Integrin alpha-6 heavy chain; Integrin alpha-6/beta-1 (ITGA6:ITGB1) is a receptor for laminin on platelets (By similarity). Integrin alpha-6/beta-1 (ITGA6:ITGB1) is present in oocytes and is involved in sperm-egg fusion (By similarity). Integrin alpha-6/beta-4 (ITGA6:ITGB4) is a receptor for laminin in epithelial cells and it plays a critical structural role in the hemidesmosome (By similarity). ITGA6:ITGB4 binds to NRG1 (via EGF domain) and this binding is essential for NRG1-ERBB signaling. ITGA6:ITGB4 binds to IGF1 and this binding is essential for IGF1 signaling. ITGA6:ITGB4 binds t [...]
78	*SLC45A1*	Proton-associated sugar transporter A; Proton-associated glucose transporter in the brain. Belongs to the glycoside-pentoside-hexuronide (GPH) cation symporter transporter (TC 2.A.2) family.
79	*ST8SIA1*	Alpha-N-acetylneuraminide alpha-2,8-sialyltransferase; Involved in the production of gangliosides GD3 and GT3 from GM3; gangliosides are a subfamily of complex glycosphinglolipds that contain one or more residues of sialic acid.
80	*RIPK2*	Receptor-interacting serine/threonine-protein kinase 2; Serine/threonine/tyrosine kinase that plays an essential role in modulation of innate and adaptive immune responses. Upon stimulation by bacterial peptidoglycans, NOD1 and NOD2 are activated, oligomerize and recruit RIPK2 through CARD-CARD domains. Contributes to the tyrosine phosphorylation of the guanine exchange factor ARHGEF2 through Src tyrosine kinase leading to NF-kappaB activation by NOD2. Once recruited, RIPK2 autophosphorylates and undergoes 'Lys-63'-linked polyubiquitination by E3 ubiquitin ligases XIAP, BIRC2 and BIRC3 [...]
81	*ATP5MC2*	ATP synthase F(0) complex subunit C2, mitochondrial; Mitochondrial membrane ATP synthase (F(1)F(0) ATP synthase or Complex V) produces ATP from ADP in the presence of a proton gradient across the membrane which is generated by electron transport complexes of the respiratory chain. F-type ATPases consist of two structural domains, F(1) - containing the extramembraneous catalytic core and F(0) - containing the membrane proton channel, linked together by a central stalk and a peripheral stalk. During catalysis, ATP synthesis in the catalytic domain of F(1) is coupled via a rotary mechanis [...]
82	*ATF7*	Cyclic AMP-dependent transcription factor ATF-7; Plays important functions in early cell signaling. Binds the cAMP response element (CRE) (consensus: 5'-GTGACGT[AG][AG]-3'), a sequence present in many viral and cellular promoters. Activator of the NF-ELAM1/delta-A site of the E-selectin promoter. Has no intrinsic transcriptional activity, but activates transcription on formation of JUN or FOS heterodimers. Also can bind

		TRE promoter sequences when heterodimerized with members of the JUN family. Isoform 5 acts as a negative regulator, inhibiting both ATF2 and ATF7 transcriptional activi [...]
83	*ATF7-NPFF*	Cyclic AMP-dependent transcription factor ATF-7; Plays important functions in early cell signaling. Binds the cAMP response element (CRE) (consensus: 5'-GTGACGT[AG][AG]-3'), a sequence present in many viral and cellular promoters.
84	*TARBP2*	RISC-loading complex subunit TARBP2; Required for formation of the RNA induced silencing complex (RISC). Component of the RISC loading complex (RLC), also known as the micro-RNA (miRNA) loading complex (miRLC), which is composed of DICER1, AGO2 and TARBP2. Within the RLC/miRLC, DICER1 and TARBP2 are required to process precursor miRNAs (pre-miRNAs) to mature miRNAs and then load them onto AGO2. AGO2 bound to the mature miRNA constitutes the minimal RISC and may subsequently dissociate from DICER1 and TARBP2. May also play a role in the production of short interfering RNAs (siRNAs) from [...]
85	*MAP3K12*	Mitogen-activated protein kinase kinase kinase 12; Part of a non-canonical MAPK signaling pathway. Activated by APOE, enhances the AP-1-mediated transcription of APP, via a MAP kinase signal transduction pathway composed of MAP2K7 and MAPK1/ERK2 and MAPK3/ERK1. May be an activator of the JNK/SAPK pathway.
86	*DGKH*	Diacylglycerol kinase eta; Phosphorylates diacylglycerol (DAG) to generate phosphatidic acid (PA). Plays a key role in promoting cell growth. Activates the Ras/B-Raf/C-Raf/MEK/ERK signaling pathway induced by EGF. Regulates the recruitment of RAF1 and BRAF from cytoplasm to membranes and their heterodimerization.
87	*KCNAB1*	Voltage-gated potassium channel subunit beta-1; Cytoplasmic potassium channel subunit that modulates the characteristics of the channel-forming alpha-subunits. Modulates action potentials via its effect on the pore-forming alpha subunits (By similarity). Promotes expression of the pore-forming alpha subunits at the cell membrane, and thereby increases channel activity (By similarity). Mediates closure of delayed rectifier potassium channels by physically obstructing the pore via its N-terminal domain and increases the speed of channel closure for other family members. Promotes the clos [...]
88	*EFHB*	EF-hand domain-containing family member B; Cytosolic sensor for calcium, modulates the interaction of STIM1 and ORAI1 upon store depletion, the activation of store-operated Ca(2+) entry (SOCE) and NFAT translocation from cytosol to nucleus.

89	*ALK*	ALK tyrosine kinase receptor; Neuronal receptor tyrosine kinase that is essentially and transiently expressed in specific regions of the central and peripheral nervous systems and plays an important role in the genesis and differentiation of the nervous system. Transduces signals from ligands at the cell surface, through specific activation of the mitogen- activated protein kinase (MAPK) pathway. Phosphorylates almost exclusively at the first tyrosine of the Y-x-x-x-Y-Y motif. Following activation by ligand, ALK induces tyrosine phosphorylation of CBL, FRS2, IRS1 and SHC1, as well as o [...]
90	*GLT1D1*	Glycosyltransferase 1 domain containing 1; Belongs to the glycosyltransferase group 1 family. Glycosyltransferase 4 subfamily.
91	*TRPC4*	Short transient receptor potential channel 4; Forms a receptor-activated non-selective calcium permeant cation channel. Acts as a cell-cell contact-dependent endothelial calcium entry channel. Probably operated by a phosphatidylinositol second messenger system activated by receptor tyrosine kinases or G- protein coupled receptors. Mediates cation entry, with an enhanced permeability to barium over calcium. May also be activated by intracellular calcium store depletion; Belongs to the transient receptor (TC 1.A.4) family. STrpC subfamily. TRPC4 sub-subfamily.
92	*EEF1AKNMT*	eEF1A lysine and N-terminal methyltransferase; Dual methyltransferase that catalyzes methylation of elongation factor 1-alpha (EEF1A1 and EEF1A2) at two different positions, and is therefore involved in the regulation of mRNA translation. Via its C-terminus, methylates EEF1A1 and EEF1A2 at the N-terminal residue 'Gly-2'. Via its N-terminus dimethylates EEF1A1 and EEF1A2 at residue 'Lys-55'. Has no activity towards core histones H2A, H2B, H3 and H4.
93	*CNTNAP2*	Contactin-associated protein-like 2; Required, with CNTNAP1, for radial and longitudinal organization of myelinated axons. Plays a role in the formation of functional distinct domains critical for saltatory conduction of nerve impulses in myelinated nerve fibers. Demarcates the juxtaparanodal region of the axo-glial junction.
94	*C1orf109*	Uncharacterized protein C1orf109; May promote cancer cell proliferation by controlling the G1 to S phase transition.
95	*NCKAP5*	NCK associated protein 5.
96	*ZNF729*	Zinc finger protein 729; May be involved in transcriptional regulation; Belongs to the krueppel C2H2-type zinc-finger protein family.
97	*ZNF98*	Zinc finger protein 98; May be involved in transcriptional regulation; Belongs to the krueppel C2H2-type zinc-finger protein family.

98	*DNMBP*	Dynamin-binding protein; Plays a critical role as a guanine nucleotide exchange factor (GEF) for CDC42 in several intracellular processes associated with the actin and microtubule cytoskeleton. Regulates the structure of apical junctions through F-actin organization in epithelial cells. Participates in the normal lumenogenesis of epithelial cell cysts by regulating spindle orientation. Plays a role in ciliogenesis (By similarity). May play a role in membrane trafficking between the cell surface and the Golgi (By similarity). ECO:0000250|UniProtKB:Q6TXD4.
99	*TRIM60*	Tripartite motif containing 60; Belongs to the TRIM/RBCC family.
100	*TRIM61*	Putative tripartite motif-containing protein 61; Tripartite motif containing 61.
101	*ZNF471*	Zinc finger protein 471; May be involved in transcriptional regulation.
102	*ZFP28*	Zinc finger protein 28 homolog; May be involved in transcriptional regulation. May have a role in embryonic development.
103	*SRGAP2*	SLIT-ROBO Rho GTPase-activating protein 2; RAC1 GTPase activating protein (GAP) that binds and deforms membranes, and regulates actin dynamics to regulate cell migration and differentiation. Plays an important role in different aspects of neuronal morphogenesis and migration mainly during development of the cerebral cortex. This includes the biogenesis of neurites, where it is required for both axons and dendrites outgrowth, and the maturation of the dendritic spines. Also stimulates the branching of the leading process and negatively regulates neuron radial migration in the cerebral c [...]
104	*GALNT18*	Polypeptide N-acetylgalactosaminyltransferase 18; Catalyzes the initial reaction in O-linked oligosaccharide biosynthesis, the transfer of an N-acetyl-D-galactosamine residue to a serine or threonine residue on the protein receptor.
105	*NELL1*	Protein kinase C-binding protein NELL1; Plays a role in the control of cell growth and differentiation. Promotes osteoblast cell differentiation and terminal mineralization.
106	*FGD4*	FYVE, RhoGEF and PH domain-containing protein 4; Activates CDC42, a member of the Ras-like family of Rho- and Rac proteins, by exchanging bound GDP for free GTP. Plays a role in regulating the actin cytoskeleton and cell shape. Activates MAPK8 (By similarity).
107	*PDE4D*	cAMP-specific 3',5'-cyclic phosphodiesterase 4D; Hydrolyzes the second messenger cAMP, which is a key regulator of many important physiological processes.
108	*SIRT2*	NAD-dependent protein deacetylase sirtuin-2; NAD-dependent protein deacetylase, which deacetylates internal lysines on histone and alpha-tubulin as well as many other proteins such as key transcription factors. Participates in the modulation of multiple and diverse biological processes such as cell cycle control, genomic integrity, microtubule dynamics, cell differentiation, metabolic networks, and autophagy. Plays a major role in the control of cell cycle progression and genomic stability. Functions in the antephase checkpoint preventing precocious mitotic entry in response to microtu [...]

109	*SHC3*	SHC-transforming protein 3; Signaling adapter that couples activated growth factor receptors to signaling pathway in neurons. Involved in the signal transduction pathways of neurotrophin-activated Trk receptors in cortical neurons.
110	*ENOX1*	Protein disulfide-thiol oxidoreductase; Probably acts as a terminal oxidase of plasma electron transport from cytosolic NAD(P)H via hydroquinones to acceptors at the cell surface. Hydroquinone oxidase activity alternates with a protein disulfide-thiol interchange/oxidoreductase activity which may control physical membrane displacements associated with vesicle budding or cell enlargement. The activities oscillate with a period length of 24 minutes and play a role in control of the ultradian cellular biological clock.
111	*HLA-DOA*	HLA class II histocompatibility antigen, DO alpha chain; Important modulator in the HLA class II restricted antigen presentation pathway by interaction with the HLA-DM molecule in B- cells. Modifies peptide exchange activity of HLA-DM.
112	*HLA-DPA1*	HLA class II histocompatibility antigen, DP alpha 1 chain; Binds peptides derived from antigens that access the endocytic route of antigen presenting cells (APC) and presents them on the cell surface for recognition by the CD4 T-cells. The peptide binding cleft accommodates peptides of 10-30 residues. The peptides presented by MHC class II molecules are generated mostly by degradation of proteins that access the endocytic route, where they are processed by lysosomal proteases and other hydrolases. Exogenous antigens that have been endocytosed by the APC are thus readily available for p [...]
113	*TMEM183A*	Transmembrane protein 183A.
114	*PPFIA4*	Liprin-alpha-4; May regulate the disassembly of focal adhesions. May localize receptor-like tyrosine phosphatases type 2A at specific sites on the plasma membrane, possibly regulating their interaction with the extracellular environment and their association with substrates (By similarity); Belongs to the liprin family. Liprin-alpha subfamily.
115	*NUFIP1*	Nuclear fragile X mental retardation-interacting protein 1; Binds RNA.
116	*SHROOM3*	Protein Shroom3; Controls cell shape changes in the neuroepithelium during neural tube closure. Induces apical constriction in epithelial cells by promoting the apical accumulation of F-actin and myosin II, and probably by bundling stress fibers (By similarity). Induces apicobasal cell elongation by redistributing gamma-tubulin and directing the assembly of robust apicobasal microtubule arrays (By similarity). Belongs to the shroom family.
117	*BEND5*	BEN domain-containing protein 5; Acts as a transcriptional repressor.

118	*AGBL4*	Cytosolic carboxypeptidase 6; Metallocarboxypeptidase that mediates deglutamylation of target proteins. Catalyzes the deglutamylation of polyglutamate side chains generated by post-translational polyglutamylation in proteins such as tubulins. Also removes polyglutamates from the carboxy-terminus of target proteins such as MYLK. Mediates deglutamylation of CGAS, regulating the antiviral activity of CGAS. Acts as a long-chain deglutamylase and specifically shortens long polyglutamate chains, while it is not able to remove the branching point glutamate, a process catalyzed by AGBL5/CCP5.
119	*MAPRE2*	Microtubule-associated protein RP/EB family member 2; May be involved in microtubule polymerization, and spindle function by stabilizing microtubules and anchoring them at centrosomes. May play a role in cell migration (By similarity); Belongs to the MAPRE family.
120	*CES5A*	Carboxylesterase 5A; Involved in the detoxification of xenobiotics and in the activation of ester and amide prodrugs.
121	*TENM4*	Teneurin-4; Involved in neural development, regulating the establishment of proper connectivity within the nervous system. Plays a role in the establishment of the anterior-posterior axis during gastrulation. Regulates the differentiation and cellular process formation of oligodendrocytes and myelination of small-diameter axons in the central nervous system (CNS). Promotes activation of focal adhesion kinase. May function as a cellular signal transducer (By similarity); Belongs to the tenascin family. Teneurin subfamily.
122	*NLRP13*	NACHT, LRR and PYD domains-containing protein 13; Involved in inflammation.
123	*TAP1*	Antigen peptide transporter 1; Involved in the transport of antigens from the cytoplasm to the endoplasmic reticulum for association with MHC class I molecules. Also acts as a molecular scaffold for the final stage of MHC class I folding, namely the binding of peptide. Nascent MHC class I molecules associate with TAP via tapasin. Inhibited by the covalent attachment of herpes simplex virus ICP47 protein, which blocks the peptide-binding site of TAP. Inhibited by human cytomegalovirus US6 glycoprotein, which binds to the lumenal side of the TAP complex and inhibits peptide translocation [...]
124	*PSMB9*	Proteasome subunit beta type-9; The proteasome is a multicatalytic proteinase complex which is characterized by its ability to cleave peptides with Arg, Phe, Tyr, Leu, and Glu adjacent to the leaving group at neutral or slightly basic pH. The proteasome has an ATP-dependent proteolytic activity. This subunit is involved in antigen processing to generate class I binding peptides. Replacement of PSMB6 by PSMB9 increases the capacity of the immunoproteasome to cleave model peptides after hydrophobic and basic residues.

125	*NTRK2*	BDNF/NT-3 growth factors receptor; Receptor tyrosine kinase involved in the development and the maturation of the central and the peripheral nervous systems through regulation of neuron survival, proliferation, migration, differentiation, and synapse formation and plasticity (By similarity). Receptor for BDNF/brain-derived neurotrophic factor and NTF4/neurotrophin-4. Alternatively can also bind NTF3/neurotrophin-3 which is less efficient in activating the receptor but regulates neuron survival through NTRK2. Upon ligand- binding, undergoes homodimerization, autophosphorylation and acti [...]
126	*LRPPRC*	Leucine-rich PPR motif-containing protein, mitochondrial; May play a role in RNA metabolism in both nuclei and mitochondria. In the nucleus binds to HNRPA1-associated poly(A) mRNAs and is part of nmRNP complexes at late stages of mRNA maturation which are possibly associated with nuclear mRNA export. May bind mature mRNA in the nucleus outer membrane. In mitochondria binds to poly(A) mRNA. Plays a role in translation or stability of mitochondrially encoded cytochrome c oxidase (COX) subunits. May be involved in transcription regulation. Cooperates with PPARGC1A to regulate certain mito [...]
127	*BCO1*	Beta,beta-carotene 15,15'-dioxygenase; Symmetrically cleaves beta-carotene into two molecules of retinal using a dioxygenase mechanism; Belongs to the carotenoid oxygenase family.
128	*SIK3*	Serine/threonine-protein kinase SIK3; Positive regulator of mTOR signaling that functions by triggering the degradation of DEPTOR, an mTOR inhibitor. Involved in the dynamic regulation of mTOR signaling in chondrocyte differentiation during skeletogenesis. Negatively regulates cAMP signaling pathway possibly by acting on CRTC2/TORC2 and CRTC3/TORC3 (Probable). Prevents HDAC4 translocation to the nucleus (By similarity); Belongs to the protein kinase superfamily. CAMK Ser/Thr protein kinase family. SNF1 subfamily.
129	*PAFAH1B2*	Platelet-activating factor acetylhydrolase IB subunit beta; Inactivates PAF by removing the acetyl group at the sn-2 position. This is a catalytic subunit.
130	*PCSK7*	Proprotein convertase subtilisin/kexin type 7; Serine endoprotease that processes various proproteins by cleavage at paired basic amino acids, recognizing the RXXX[KR]R consensus motif. Likely functions in the constitutive secretory pathway.
131	*CLEC16A*	Protein CLEC16A; Regulator of mitophagy through the upstream regulation of the RNF41/NRDP1-PRKN pathway. Mitophagy is a selective form of autophagy necessary for mitochondrial quality control. The RNF41/NRDP1-PRKN pathway regulates autophagosome-lysosome fusion during late mitophagy. May protect RNF41/NRDP1 from proteosomal degradation, RNF41/NRDP1 which regulates proteosomal degradation of PRKN. Plays a key role in beta cells functions by regulating mitophagy/autophagy and mitochondrial health; Belongs to the CLEC16A/gop-1 family.
132	*CNPY1*	Canopy FGF signaling regulator 1; Belongs to the canopy family.

133	*CECR2*	Cat eye syndrome critical region protein 2; Chromatin reader component of histone-modifying complexes, such as the CERF (CECR2-containing-remodeling factor) complex and ISWI- type complex. It thereby plays a role in various processes during development: required during embryogenesis for neural tube closure and inner ear development. In adults, required for spermatogenesis, via the formation of ISWI-type chromatin complexes (By similarity). In histone-modifying complexes, CECR2 recognizes and binds acylated histones: binds histones that are acetylated and/or butyrylated. May also be inv [...]
134	*RASGEF1B*	Ras-GEF domain-containing family member 1B; Guanine nucleotide exchange factor (GEF) with specificity for RAP2A, it doesn't seems to activate other Ras family proteins (in vitro).
135	*ADGRB3*	Adhesion G protein-coupled receptor B3; Receptor that plays a role in the regulation of synaptogenesis and dendritic spine formation at least partly via interaction with ELMO1 and RAC1 activity (By similarity). Promotes myoblast fusion through ELMO/DOCK1. Belongs to the G-protein coupled receptor 2 family. Adhesion G-protein coupled receptor (ADGR) subfamily.
136	*BARX1*	Homeobox protein BarH-like 1; Transcription factor, which is involved in craniofacial development, in odontogenesis and in stomach organogenesis. May have a role in the differentiation of molars from incisors. Plays a role in suppressing endodermal Wnt activity (By similarity). Binds to a regulatory module of the NCAM promoter.
137	*PTPDC1*	Protein tyrosine phosphatase domain-containing protein 1; May play roles in cilia formation and/or maintenance. Belongs to the protein-tyrosine phosphatase family. Non- receptor class PTPDC1 subfamily.
138	*HOMER1*	Homer protein homolog 1; Postsynaptic density scaffolding protein. Binds and cross- links cytoplasmic regions of GRM1, GRM5, ITPR1, DNM3, RYR1, RYR2, SHANK1 and SHANK3. By physically linking GRM1 and GRM5 with ER- associated ITPR1 receptors, it aids the coupling of surface receptors to intracellular calcium release. May also couple GRM1 to PI3 kinase through its interaction with AGAP2. Isoform 1 regulates the trafficking and surface expression of GRM5. Isoform 3 acts as a natural dominant negative, in dynamic competition with constitutively expressed isoform 1 to regulate synaptic meta [...]
139	*ZFPM2*	Zinc finger protein ZFPM2; Transcription regulator that plays a central role in heart morphogenesis and development of coronary vessels from epicardium, by regulating genes that are essential during cardiogenesis. Essential cofactor that acts via the formation of a heterodimer with transcription factors of the GATA family GATA4, GATA5 and GATA6. Such heterodimer can both activate or repress transcriptional activity, depending on the cell and promoter context. Also required in gonadal differentiation, possibly be regulating expression of SRY. Probably acts a corepressor of NR2F2 (By sim [...]

140	*PHKG2*	Phosphorylase b kinase gamma catalytic chain, liver/testis isoform; Catalytic subunit of the phosphorylase b kinase (PHK), which mediates the neural and hormonal regulation of glycogen breakdown (glycogenolysis) by phosphorylating and thereby activating glycogen phosphorylase. May regulate glycogeneolysis in the testis. In vitro, phosphorylates PYGM (By similarity); Belongs to the protein kinase superfamily. CAMK Ser/Thr protein kinase family.
141	*XRN2*	5'-3' exoribonuclease 2; Possesses 5'->3' exoribonuclease activity (By similarity). May promote the termination of transcription by RNA polymerase II. During transcription termination, cleavage at the polyadenylation site liberates a 5' fragment which is subsequently processed to form the mature mRNA and a 3' fragment which remains attached to the elongating polymerase. The processive degradation of this 3' fragment by this protein may promote termination of transcription. Binds to RNA polymerase II (RNAp II) transcription termination R-loops formed by G- rich pause sites. Belongs to t [...]
142	*RYR1*	Ryanodine receptor 1; Calcium channel that mediates the release of Ca(2+) from the sarcoplasmic reticulum into the cytoplasm and thereby plays a key role in triggering muscle contraction following depolarization of T-tubules. Repeated very high-level exercise increases the open probability of the channel and leads to Ca(2+) leaking into the cytoplasm. Can also mediate the release of Ca(2+) from intracellular stores in neurons, and may thereby promote prolonged Ca(2+) signaling in the brain. Required for normal embryonic development of muscle fibers and skeletal muscle. Required for nor [...]
143	*ALG11*	GDP-Man:Man(3)GlcNAc(2)-PP-Dol alpha-1,2-mannosyltransferase; Mannosyltransferase involved in the last steps of the synthesis of Man5GlcNAc(2)-PP-dolichol core oligosaccharide on the cytoplasmic face of the endoplasmic reticulum. Catalyzes the addition of the 4th and 5th mannose residues to the dolichol-linked oligosaccharide chain; Belongs to the glycosyltransferase group 1 family. Glycosyltransferase 4 subfamily.
144	*NEK5*	Serine/threonine-protein kinase Nek5; NIMA related kinase 5.
145	*NEK3*	Serine/threonine-protein kinase Nek3; Protein kinase which influences neuronal morphogenesis and polarity through effects on microtubules. Regulates microtubule acetylation in neurons. Contributes to prolactin-mediated phosphorylation of PXN and VAV2. Implicated in prolactin-mediated cytoskeletal reorganization and motility of breast cancer cells through mechanisms involving RAC1 activation and phosphorylation of PXN and VAV2.
146	*PPP6R2*	Serine/threonine-protein phosphatase 6 regulatory subunit 2; Regulatory subunit of protein phosphatase 6 (PP6). May function as a scaffolding PP6 subunit. Involved in the PP6-mediated dephosphorylation of NFKBIE opposing its degradation in response to TNF-alpha; Belongs to the SAPS family.

147	*PTGER3*	Prostaglandin E2 receptor EP3 subtype; Receptor for prostaglandin E2 (PGE2). The activity of this receptor can couple to both the inhibition of adenylate cyclase mediated by G(i) proteins, and to an elevation of intracellular calcium. Required for normal development of fever in response to pyrinogens, including IL1B, prostaglandin E2 and bacterial lipopolysaccharide (LPS). Required for normal potentiation of platelet aggregation by prostaglandin E2, and thus plays a role in the regulation of blood coagulation. Required for increased HCO3(-) secretion in the duodenum in response to muco [...]
148	*DNAJC12*	DnaJ heat shock protein family member C12.
149	*SELENOO*	Protein adenylyltransferase SelO, mitochondrial; Catalyzes the transfer of adenosine 5'-monophosphate (AMP) to Ser, Thr and Tyr residues of target proteins (AMPylation). May be a redox-active mitochondrial selenoprotein which interacts with a redox target protein. Belongs to the SELO family.

Supplementary Table S3. Long noncoding RNA genes associated with hypertension in the DISFIN Study.

No	SNV	Gene	EUR_AF	DF_AF	CADD	Remarks
1	rs7200229	*FLYWCH1-AS1*	0.6799	0.3621	0.552	
2	rs675828	*ENSG00000294624*	0.5467	0.4093	0.219	
3	rs12472254	*LINC01320*	0.2932	0.215	0.183	
4	rs10075131	*ENSG00000249776*	0.5487	0.4775	22.2	Associated with Diastolic blood pressure (ExPheWas database)
5	rs4611977	*ENSG00000287571*	0.5398	0.4193	0.464	
6	rs4779012	*ANKRD34C-AS1*	0.4324	0.3208	0.502	Associated with Diastolic blood pressure (T2DKP database)
7	rs12025839	*VAV3-AS1*	0.2416	0.2531	1.176	Regulates the VAV3 gene which controls vascular tone. Is associated with risk of hypertension [1]
8	rs6432051	*ENSG00000309032*	0.16	0.332	0.043	
9	rs73023553	*LINC00574*	0.1481	0.2017	4.899	
10	rs9328336	*ENSG00000300014*	0.5765	0.3955	1.372	
11	rs10885198	*ENSG00000307856*	0.4811	0.4702	8.372	Associated with arterial stiffness and diastolic blood pressure, Framingham Heart Study 100K Project [2]
12	rs2654735	*ENSG00000248656*	0.4294	0.3406	0.987	
13	rs62105283	*ENSG00000264015*	0.0686	0.1408	3.54	Associated with Diastolic blood pressure (ExPheWas database)
14	rs7697417	*ENSG00000285454*	0.3767	0.3176	1.161	
15	rs1412294	*ENSG00000285637*	0.3062	0.4568	2.597	
16	rs12585338	*LINC00540*	0.0139	0.0989	3.962	
17	rs13359266	*ENSG00000248605*	0.1938	0.2869	2.101	
18	rs2048265	*LINC02516*	0.5686	0.2748	3.169	
19	rs12709705	*ENSG00000294453*	0.1034	0.2082	1.348	

20	rs58074807	*MGC4859*	0.2694	0.3226	5.72	Associated with risk of hypertension and contributes to sex disparity in hypertension[3]
21	rs7894962	*ENSG00000232985*	0.3897	0.2876	9.481	
22	rs12468912	*ENSG00000286979*	0.498	0.4216	5.238	Associated with Systolic and diastolic blood pressure and with risk of hypertension (Common Metabolic Diseases Knowledge Portal)
23	rs10798510	*ENSG00000305548*	0.6511	0.407	4.958	
24	rs378495	*ENSG00000305367*	0.6431	0.4461	0.346	
25	rs1005060	*VPS33B-DT*	0.4334	0.4436	2.472	
26	rs62191170	*LINC01237*	0.6918	0.1784	1.872	Associated with risk of hypertension[4]
27	rs10816605	*ENSG00000303954*	0.1004	0.3286	3.68	
28	rs79245693	*CCNT2-AS1*	0.2266	0.2636	3.578	Associated with blood pressure regulation[5]
29	rs717519	*ENSG00000302585*	0.6014	0.4791	1.635	
30	rs2223930	*LINC02306*	0.2406	0.4414	1.02	
31	rs17595186	*ENSG00000272777*	0.2843	0.1499	2.969	
32	rs12615167	*ENSG00000293776*	0.498	0.3621	0.994	
33	rs11041816	*ENSG00000307368*	0.4712	0.4478	18.52	
34	rs11204103	*ENSG00000309971*	0.4235	0.4138	0.68	
35	rs7538519	*ENSG00000287364*	0.1183	0.2767	1.277	
36	rs12443114	*LINC02253*	0.2674	0.3914	1.396	
37	rs13236240	*ENSG00000287860*	0.5666	0.4775	5.931	Systolic and Diastolic blood pressure (Enhancer2Gene database, Stanford University)
38	rs11942693	*ENSG00000250887*	0.1769	0.2767	0.083	

39	rs530271	*SNAP25-AS1*	0.4016	0.4776	5.438	Associated with blood pressure regulation[6]
40	rs4948446	*LINC00845*	0.5447	0.3673	0.909	Associated with Systolic blood pressure (GWAS Catalog)
41	rs4942380	*ENSG00000298544*	0.7803	0.3568	2.517	
42	rs3828893	*MICB-DT*	0.0964	0.0486	1.156	Associated with Systolic blood pressure (Common Metabolic Diseases Knowledge Portal)
43	rs7317149	*ENSG00000285572*	0.8241	0.2128	3.669	Associated with Diastolic blood pressure (Common Metabolic Diseases Knowledge Portal)
44	rs1147199	*ENSG00000285987*	0.8161	0.3173	2.445	
45	rs7120515	*ENSG00000224077*	0.6402	0.3511	4.504	
46	rs4281012	*STAG3L4*	0.1322	0.2286	0.914	Some databases list this as a pseudogene
47	rs154802	*ENSG00000249984*	0.8559	0.2683	0.356	
48	rs11793324	*BARX1-DT*	0.1044	0.1247	12.66	
49	rs7894961	*ENSG00000296083*	0.2207	0.4273	5.166	
50	rs10858101	*PPP1R26-AS1*	0.7276	0.4081	4.088	
51	rs12637115	*ENSG00000223727*	0.6103	0.3464	0.295	Associated with Diastolic blood pressure (T2DKP database)
52	rs6035868	*ENSG00000225280*	0.5855	0.3582	13.05	
53	rs150593977	*AHI1-DT*	0	0.0177	1.9	Associated with Systlic and Diastolic blood pressure (Common Metabolic Diseases Knowledge Portal)
54	rs78107974	*ENSG00000230027*	0.0258	0.0147	0.156	Associated with blood pressure via effects on Fibroblast Growth Factor 1[7-9]

55	rs77574509	*ENSG00000225233*	0	0.0239	1.994	Associated with Systolic and diastolic blood pressure and with risk of hypertension (Common Metabolic Diseases Knowledge Portal)

Supplementary Table S4. Human Phenotype Ontology terms related to significantly associated genes in the DISFIN Study.

HPO Term	Description	p	FDRp	N	B	Gene Symbol
EFO:0004557	Population measurement	5.8E-11	7E-07	24	607	*SBF2, CCNT2, RASGEF1B, NTRK2, TENM4, GLT1D1, SHROOM3, PCLO, PTGER3, CNTNAP2, PCDH15, SIK3, MAP3K19, DPP10, ZFPM2, CLEC16A, NCKAP5, RBFOX1, FTO, CCSER1, CES5A, PLD5, CSMD1, LRP2*
EFO:0007874	Gut microbiome measurement	2.1E-10	1E-06	21	485	*CCNT2, RASGEF1B, NTRK2, TENM4, GLT1D1, SHROOM3, PCLO, PTGER3, CNTNAP2, PCDH15, MAP3K19, DPP10, ZFPM2, CLEC16A, NCKAP5, RBFOX1, CCSER1, CES5A, PLD5, CSMD1, LRP2*
EFO:0004302	Anthropometric measurement	3.0E-09	9E-06	65	4304	*SBF2, LRPPRC, EPAS1, ADAMTS17, NTRK2, TENM4, NELL1, ZNF471, KCNH5, PCSK7, GALNT17, SPSB1, TRDN, HOMER1, DGKH, DUPD1, NLRP13, PDE4D, ARHGAP12, MAGI2, TAP1, SYT17, ACMSD, ELAVL4, RYR1, CYB5R3, CNTNAP2, HBS1L, TCERG1L, ADGRB3, BEND5, AGBL4, PCDH15, LIN28A, SUSD1, PSMB9, SIK3, XRN2, ALK, ARHGAP8, DPP10, ANKRD13B, ST8SIA1, ABCC1, CECR2, ZFPM2, A4GALT, CLEC16A, NCKAP5, RBFOX1, HLA-DPA1, ATF7, SLC45A1, FTO, KCNAB1, CCSER1, SELENOP, PAFAH1B2, MAP3K12, ATF7-NPFF, GHR, TRPC4, CSMD1, SLC6A1, ATP5MC2*

EFO:0004324	Body weights and measures	1.1E-08	3E-05	62	4122	*SBF2, EPAS1, ADAMTS17, NTRK2, TENM4, NELL1, ZNF471, KCNH5, PCSK7, GALNT17, SPSB1, TRDN, HOMER1, DGKH, DUPD1, NLRP13, PDE4D, ARHGAP12, MAGI2, TAP1, SYT17, ACMSD, ELAVL4, CYB5R3, CNTNAP2, HBS1L, TCERG1L, ADGRB3, BEND5, AGBL4, PCDH15, LIN28A, SUSD1, PSMB9, SIK3, XRN2, ALK, ARHGAP8, DPP10, ANKRD13B, ST8SIA1, ABCC1, CECR2, ZFPM2, A4GALT, CLEC16A, NCKAP5, RBFOX1, HLA-DPA1, ATF7, FTO, KCNAB1, CCSER1, SELENOP, PAFAH1B2, MAP3K12, ATF7-NPFF, GHR, TRPC4, CSMD1, SLC6A1, ATP5MC2*
EFO:0004464	Brain measurement	2.7E-07	0.0006	27	1174	*GALNT18, SBF2, RNFT2, NUAK1, ADD2, ADAMTS17, TENM4, HOMER1, PDE4D, RYR1, CNTNAP2, ADGRB3, AGBL4, SHC3, SELENOO, ANKRD13B, ABCC1, CLEC16A, NCKAP5, RBFOX1, SPATA13, ATF7, SRGAP2, ATF7-NPFF, CSMD1, FAM240C, LRP2*
EFO:0006848	Mental or behavioural disorder biomarker	1.5E-06	0.0025	31	1615	*GALNT18, PTGIS, SBF2, LRPPRC, NUAK1, ENOX1, TENM4, MAPRE2, ZNF609, PIF1, GALNT17, TRIM61, TRDN, PCLO, DGKH, MAGI2, PTGER3, RYR1, CYB5R3, CNTNAP2, TCERG1L, ADGRB3, AGBL4, SIK3, DPP10, ZFPM2, CLEC16A, RBFOX1, FTO, CCSER1, CSMD1*
EFO:0004747	Protein measurement	3.5E-06	0.0053	71	5856	*DNAJC12, SIRT2, SBF2, RNFT2, BCO1, TMCC3, ENOX1, EPAS1, NTRK2, TENM4, PPP1R21, SHROOM3, NELL1, MAPRE2, ZFP28, DNMBP, ZNF609, CNPY1, IL23R, KCNH5, PCSK7, GALNT17, TRIM61, TRDN, HOMER1, DGKH, PDE4D, MAGI2, ACMSD, GRM7, ZNF98, RYR1, CNTNAP2, HBS1L, TCERG1L, ADGRB3, C1orf141, AGBL4, PCDH15, SUSD1, PSMB9, SIK3, SHC3, XRN2, NUFIP1, CDH18, ALK, MAP3K19, ABCC1, ZFPM2, A4GALT, CLEC16A, NCKAP5, RBFOX1, HLA-DPA1, SPATA13, ATF7, SLC45A1, FTO, CCSER1, SELENOP, PAFAH1B2, PLD5, SRGAP2, ATF7-NPFF, PPP6R2, GHR, TRPC4, CSMD1, LRP2, TRIM60*

EFO:0004730	Hormone measurement	4.2E-06	0.0057	29	1527	*SBF2, TMCC3, RASGEF1B, PPP1R21, SHROOM3, NELL1, DNMBP, DGKH, PDE4D, ACMSD, GRM7, CNTNAP2, ADGRB3, AGBL4, PCDH15, SUSD1, NUFIP1, ALK, MAP3K19, DPP10, ZFPM2, CLEC16A, RBFOX1, SPATA13, FTO, SELENOP, PLD5, GHR, CSMD1*
EFO:0003892	Pulmonary function measurement	5.1E-06	0.0062	25	1208	*SBF2, ENOX1, GLT1D1, SHROOM3, SDC2, GALNT17, TRDN, PCLO, DGKH, PDE4D, MAGI2, CNTNAP2, AGBL4, PCDH15, LIN28A, DPP10, ST8SIA1, CLEC16A, SPATA13, FTO, SRGAP2, PPP6R2, CSMD1, SLC6A1, LRP2*
EFO:0000719	Temporal measurement	1.1E-05	0.012	26	1351	*SBF2, VIPR2, CCNT2, RASGEF1B, GLT1D1, SHROOM3, PDE4D, MAGI2, NEK5, GRM7, CNTNAP2, TCERG1L, C1orf141, PCDH15, SUSD1, CDH18, ALK, DPP10, CLEC16A, NCKAP5, RBFOX1, HLA-DPA1, FTO, PLD5, CSMD1, LRP2*
EFO:0004338	Body weight	1.3E-05	0.012	12	337	*EPAS1, TENM4, DUPD1, NLRP13, PCDH15, ARHGAP8, DPP10, ZFPM2, RBFOX1, FTO, KCNAB1, CCSER1*
EFO:0004556	Antibody measurement	1.1E-05	0.012	12	330	*EPAS1, DGKH, NEK5, PTGER3, CNTNAP2, RBFOX1, HLA-DPA1, UNC13A, ALG11, PLD5, CSMD1, TRIM60*
EFO:0006842	Diabetes mellitus biomarker	1.3E-05	0.012	17	658	*SBF2, TMCC3, TENM4, GRM7, HBS1L, AGBL4, PCDH15, SIK3, SHC3, ALK, MAP3K19, ABCC1, ZFPM2, FTO, CCSER1, PLD5, CSMD1*
EFO:0008111	Diet measurement	1.3E-05	0.012	10	227	*NELL1, MAGI2, SYT17, ZNF98, CNTNAP2, ADGRB3, RBFOX1, FTO, CCSER1, CSMD1*
EFO:0007010	Drug use measurement	1.6E-05	0.0121	13	403	*SBF2, LRPPRC, NELL1, KCNH5, C1QTNF6, RIMKLB, CNTNAP2, SIK3, ZFPM2, CLEC16A, SPATA13, FTO, CCSER1*

EFO:0004325	Blood pressure	1.9E-05	0.0132	23	1138	*GALNT18, SBF2, VIPR2, TENM4, SHROOM3, DUSP16, ZNF609, DGKH, ACMSD, GRM7, ZNF98, AGBL4, SIK3, CDH18, ALK, ZFPM2, RBFOX1, FTO, FGD4, PAFAH1B2, TRPC4, CSMD1, LRP2*
EFO:0004460	Soluble transferrin receptor measurement	2.3E-05	0.0132	3	5	*PCSK7, SIK3, PAFAH1B2*
EFO:0006527	Smoking status measurement	2.0E-05	0.0132	15	541	*MAGI2, PTGER3, ELAVL4, GRM7, ZNF98, CNTNAP2, ADGRB3, AGBL4, PCDH15, ALK, ST8SIA1, RBFOX1, FTO, CCSER1, TRPC4*
EFO:0006930	Brain volume measurement	1.9E-05	0.0132	16	608	*RNFT2, NUAK1, ADD2, ADAMTS17, HOMER1, PDE4D, CNTNAP2, ADGRB3, AGBL4, SHC3, SELENOO, CLEC16A, NCKAP5, RBFOX1, CSMD1, FAM240C*
EFO:0007645	Longitudinal alcohol consumption measurement	2.0E-05	0.0132	4	18	*LRPPRC, PTGER3, FTO, SLC6A1*
EFO:0004340	Body mass index	3.0E-05	0.0156	24	1257	*ADAMTS17, NTRK2, NELL1, KCNH5, TRDN, MAGI2, ELAVL4, CNTNAP2, TCERG1L, ADGRB3, BEND5, AGBL4, PCDH15, SIK3, ALK, ARHGAP8, ZFPM2, NCKAP5, RBFOX1, FTO, CCSER1, PAFAH1B2, MAP3K12, CSMD1*
EFO:0005134	Amino acid measurement	3.3E-05	0.0166	11	311	*SHROOM3, NELL1, IL23R, TRDN, ACMSD, C1orf141, PCDH15, ALK, RBFOX1, CCSER1, CSMD1*
EFO:0005671	Smoking behaviour measurement	4.1E-05	0.0194	16	649	*MAGI2, PTGER3, ELAVL4, GRM7, ZNF98, CNTNAP2, ADGRB3, AGBL4, PCDH15, ALK, ST8SIA1, RBFOX1, FTO, CCSER1, TRPC4, LRP2*
EFO:0006335	Systolic blood pressure	4.0E-05	0.0194	19	872	*SBF2, TENM4, SHROOM3, DUSP16, ZNF609, DGKH, GRM7, ZNF98, AGBL4, SIK3, CDH18, ALK, ZFPM2, RBFOX1, FTO, FGD4, PAFAH1B2, CSMD1, LRP2*

EFO:0004529	Lipid measurement	4.4E-05	0.0198	36	2400	*SBF2, ADAMTS17, TENM4, SHROOM3, NELL1, DNMBP, BASP1, IL23R, PCSK7, DGKH, PDE4D, MAGI2, ACMSD, GRM7, ZNF98, CNTNAP2, EEF1AKNMT, HBS1L, C1orf141, PCDH15, SUSD1, SIK3, NUFIP1, ALK, DPP10, ZFPM2, A4GALT, NCKAP5, RBFOX1, FTO, CCSER1, PAFAH1B2, PLD5, SRGAP2, PPP6R2, CSMD1*
EFO:0005105	Lipid or lipoprotein measurement	5.5E-05	0.024	37	2526	*SBF2, ADAMTS17, TENM4, SHROOM3, NELL1, ZFP28, DNMBP, BASP1, IL23R, PCSK7, DGKH, PDE4D, MAGI2, ACMSD, GRM7, ZNF98, CNTNAP2, EEF1AKNMT, HBS1L, C1orf141, PCDH15, SUSD1, SIK3, NUFIP1, ALK, DPP10, ZFPM2, A4GALT, NCKAP5, RBFOX1, FTO, CCSER1, PAFAH1B2, PLD5, SRGAP2, PPP6R2, CSMD1*
EFO:0006946	Behavioural disinhibition measurement	5.6E-05	0.024	6	83	*TCERG1L, ADGRB3, ZFPM2, FTO, CCSER1, CSMD1*
EFO:0000246	Age	6.0E-05	0.0245	17	745	*VIPR2, CCNT2, RASGEF1B, GLT1D1, PDE4D, MAGI2, GRM7, PCDH15, CDH18, ALK, CLEC16A, NCKAP5, RBFOX1, HLA-DPA1, FTO, PLD5, CSMD1*
EFO:0004516	Bone fracture related measurement	1.2E-04	0.044	23	1289	*PTGIS, SBF2, ADAMTS17, NELL1, ZNF471, BASP1, RYR1, CNTNAP2, PCDH15, LIN28A, A4GALT, RBFOX1, SPATA13, ATF7, SLC45A1, FTO, CCSER1, SELENOP, ATF7-NPFF, PTPDC1, PPP6R2, GHR, ATP5MC2*
EFO:0004530	Triglyceride measurement	1.2E-04	0.044	17	787	*ADAMTS17, TENM4, SHROOM3, NELL1, PCSK7, GRM7, CNTNAP2, EEF1AKNMT, PCDH15, SIK3, ALK, NCKAP5, RBFOX1, FTO, PAFAH1B2, PLD5, CSMD1*

EFO:0004725	Metabolite measurement	1.2E-04	0.044	20	1033	*SBF2, NTRK2, SHROOM3, NELL1, DGKH, MAGI2, SYT17, ACMSD, GRM7, CNTNAP2, PCDH15, ABCC1, ZFPM2, A4GALT, RBFOX1, ATF7, CCSER1, ATF7-NPFF, SLC6A1, LRP2*
EFO:0004870	Sleep measurement	1.2E-04	0.044	18	868	*ADAMTS17, ST8SIA2, TENM4, KCNH5, PCSK7, DUPD1, PDE4D, MAGI2, MYOM1, GRM7, PCDH15, SIK3, DPP10, ZFPM2, RBFOX1, FTO, CCSER1, CSMD1*
EFO:0004847	Age at onset	1.3E-04	0.0453	10	304	*VIPR2, RASGEF1B, GLT1D1, GRM7, CDH18, CLEC16A, NCKAP5, RBFOX1, HLA-DPA1, PLD5*

Supplementary Figure S1. Variant-phenotype network using data from GWASCatalog.

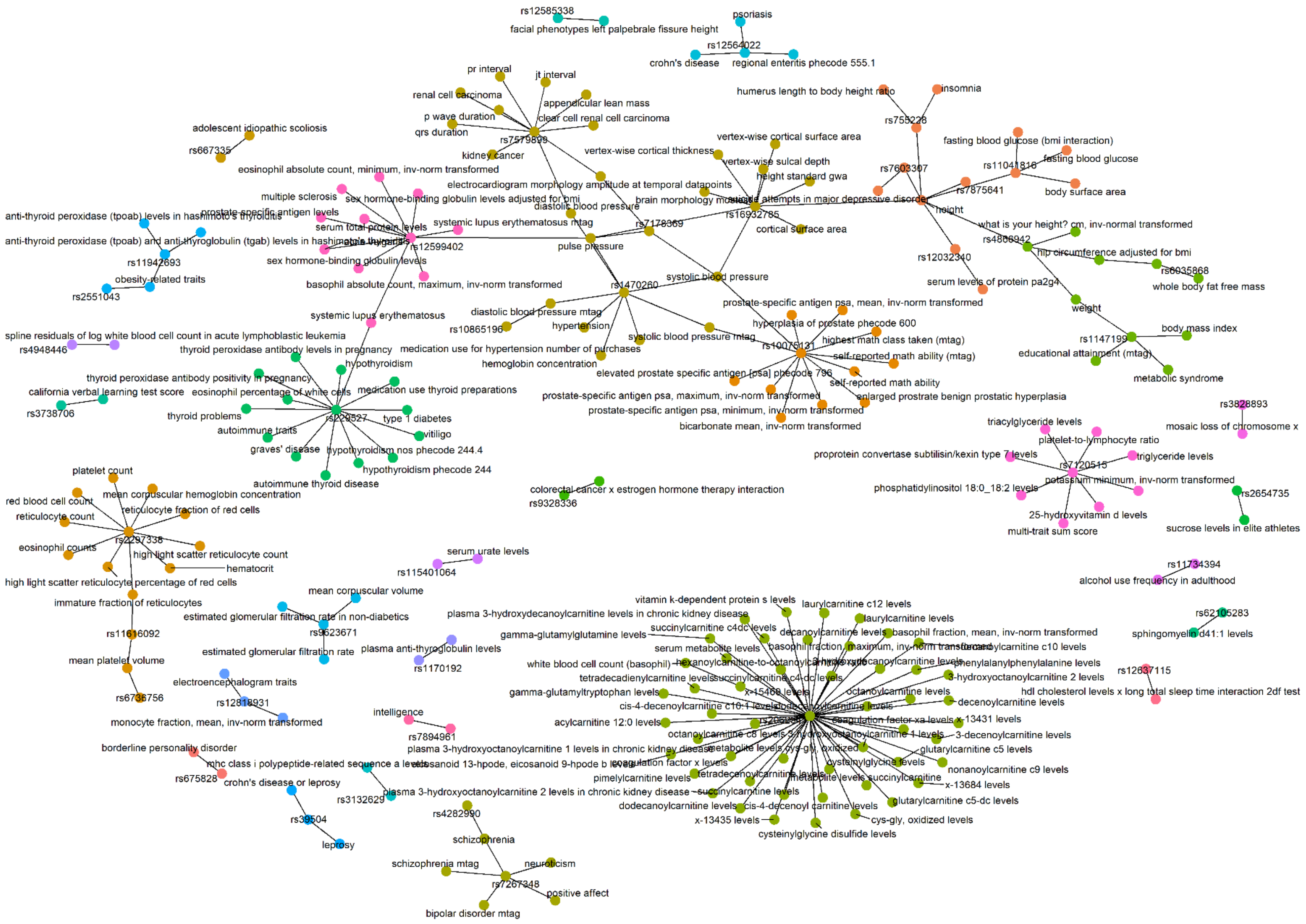

Supplementary Figure S2. Gene set enrichment analyses using the g:Profiler tool. Statistically significant terms are listed and detailed in the inset table. The gene ontology term identifiers in the table match those in the chart. Source databases used are: GO:MF, Gene ontology molecular function; GO:BP, Gene ontology biological process; GO:CC, Gene ontology cellular component; KEGG, Kyoto Encyclopedia of Genes and Genomes; REAC, Reactome; WP, WikiPathways; MIRNA, miRTarBase; HPA, Human Protein Atlas; CORUM, Comprehensive resource of mammalian protein complexes; HP, Human Phenotype Ontology

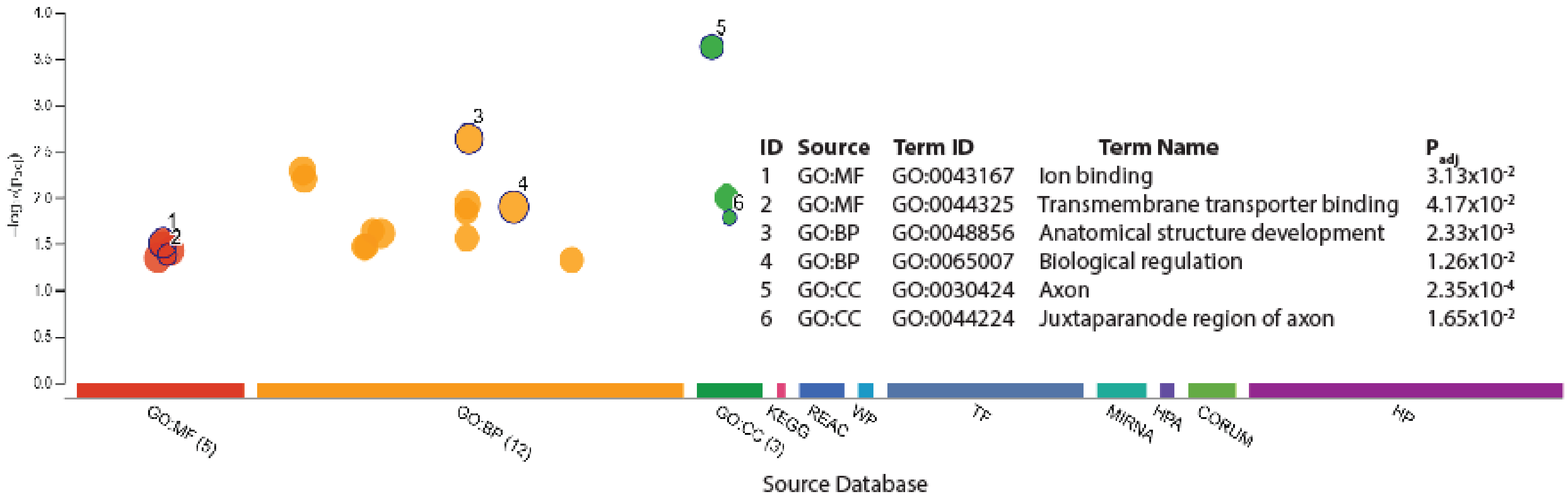

ID	Source	Term ID	Term Name	P_{adj}
1	GO:MF	GO:0043167	Ion binding	3.13×10^{-2}
2	GO:MF	GO:0044325	Transmembrane transporter binding	4.17×10^{-2}
3	GO:BP	GO:0048856	Anatomical structure development	2.33×10^{-3}
4	GO:BP	GO:0065007	Biological regulation	1.26×10^{-2}
5	GO:CC	GO:0030424	Axon	2.35×10^{-4}
6	GO:CC	GO:0044224	Juxtaparanode region of axon	1.65×10^{-2}

Supplementary Note S1: R code for conducting GWAS

```
# Set the working directory to the specified path where data files are located
setwd("Path/to/the/working/directory")

# Load the sample pool data from a CSV file into a data frame called 'pools'
pools <- read.csv("sample_pool.csv", header=TRUE)
# Load allele frequency data from a CSV file into a data frame called 'afdata'
afdata <- read.csv("AFData.csv", header=TRUE)

# Load the 'survey' package, which provides functions for survey analysis
library(survey)

# Initialize empty vectors to store results from the loop
p <- vector()      # To store p-values
maf <- vector()    # To store minor allele frequencies (MAF)
t <- vector()      # To store t-statistics
coef <- vector()   # To store coefficients from the regression model
sd <- vector()     # To store standard deviations of the coefficients
csq <- vector()    # To store chi-squared statistics

# Loop over each SNP (Single Nucleotide Polymorphism) from 1 to 665608
for (sn in c(1:665608)) {

# for (sn in c(1:20)) {

  # Calculate the B-allele frequency (BAF) for the current SNP using data from
'afdata'
  pools$baf <- 100 * t(afdata[sn, pools$Colno])
  names(pools)[14] <- "baf"  # Rename the 14th column of 'pools' to 'baf'

  # If there are no missing values in the 'baf' column
  if (sum(is.na(pools$baf)) == 0) {

  # Define a survey design object with PoolID as the cluster variable and 'n' as the
weights
    pl_svy <- svydesign(ids = ~PoolID, weights = ~n, data = pools)

    # Fit a logistic regression model (quasibinomial family) with the variables of
interest
    # lrfit <- svyglm(t2d ~ baf + Replicate + batch + mean_phi + cob + htn + dl + (1 |
PoolID), pl_svy, family = "quasibinomial")
    lrfit <- svyglm(htn ~ baf + Replicate + batch + mean_phi + cob + t2d + dl + (1 |
PoolID), pl_svy, family = "quasibinomial")

    # Store the coefficient of 'baf' (SNP effect) and its standard deviation
    coef[sn] <- lrfit$coefficients[2]
    sd[sn] <- sqrt(lrfit$cov.unscaled[2,2])

    # Calculate the t-statistic for the coefficient
    t[sn] <- coef[sn] / sd[sn]

    # Calculate the two-tailed p-value for the t-statistic
    p[sn] <- 2 * pt(abs(t[sn]), 19, lower.tail = FALSE)

    # Calculate the chi-squared statistic from the p-value
    csq[sn] <- qchisq(p[sn], 1, lower.tail = FALSE)

    # Calculate the minor allele frequency (MAF) across pools
    m <- 0
    for (pl in c(1:14)) {
      m <- m + (pools$pool.F[(pl-1)*2+1] * pools$baf[(pl-1)*2+1])
    }
```

```
    if (m > 50) {
      m <- 100 - m  # Ensure MAF is less than or equal to 50%
    }
    maf[sn] <- m  # Store the calculated MAF

  } else {
    # If there are missing values, store NA for all corresponding statistics
    p[sn] <- NA
    maf[sn] <- NA
    t[sn] <- NA
    coef[sn] <- NA
    sd[sn] <- NA
    csq[sn] <- NA
  }
}
```

Supplementary Note S2. R code for adjusting p values

```
# Load necessary libraries
library(dplyr)

# Set the working directory
setwd("Path/to/the/working/directory")

# Read in the CSV file
gwas1 <- read.csv("Final Results - HTN.csv")
cat("1:", nrow(gwas1))

# Ensure p values are between 0 and 1, and exclude NAs
gwas1 <- gwas1[!is.na(gwas1$p) & gwas1$p > 0 & gwas1$p <= 1, ]
cat("2: ", nrow(gwas1))

hist(gwas1$p)
median(gwas1$p)

# Exclude records based on specified criteria
gwas1 <- gwas1 %>%
  filter(Address != '0' & Address != "",          # Exclude if Address is '0' or empty
         !Chr %in% c("0", "X", "Y", "MT", "XY"),       # Exclude if Chr is X, Y, or MT
         maf >= 0.01)                             # Exclude if maf is less than 0.01

table(gwas1$Chr)

cat("3: ", nrow(gwas1))  # Check number of rows after filtering

## Genomic control correction first, BH correction later ##

# Apply genomic control correction
chisq <- qchisq(gwas1$p, df = 1, lower.tail = FALSE)
#lambda <- median(chisq) / qchisq(0.5, df = 1)     # Calculate lambda
lambda <- qchisq(median(gwas1$p), df=1) / qchisq(0.5, df = 1)
cat("Lambda value for genomic control correction:", lambda, "\n")  # Print lambda
cat("median p value: ", median(gwas1$p))
cat("denom: ", qchisq(0.5, df = 1))
cat("numer: ", median(chisq))
cat("chiq median:", median(gwas1$csq))

newchisq <- chisq / lambda
gwas1$P_new <- pchisq(newchisq, df = 1, lower.tail = FALSE)

# Apply BH correction
gwas1$P_BH <- p.adjust(gwas1$P_new, method = "BH")

# Sort data by P_BH in ascending order
gwas1 <- gwas1 %>%
  arrange(P_BH)

# Write the sorted data frame to a new CSV file
write.csv(gwas1, "Final Results - HTN_corrected_BH.csv", row.names = FALSE)

# Filter records where P_BH < 0.05 and output to a separate file
gwas1_top <- gwas1 %>%
  filter(P_BH < 0.05)

write.csv(gwas1_top, "PBH_topN.csv", row.names = FALSE)
```

Supplementary Note S3. R code for creating plots

```
# Load necessary libraries
library(dplyr)  # For data manipulation
library(qqman)  # For QQ and Manhattan plots

# Read in the CSV file
data <- read.csv("Final Results - HTN_corrected_BH.csv")
data <- data[!(data$Chr %in% c("X", "Y", "XY")), ]  # Remove rows with X, Y, XY
# Check for any non-numeric chromosome names (like 'X' or 'Y')
unique(data$Chr)

# Identify and remove rows with NA values
if (anyNA(data)) {
  cat("NA values detected in the dataset. Removing rows with NA values...\n")
  data <- na.omit(data)
  cat("Rows with NA values have been removed.\n")
} else {
  cat("No NA values detected in the dataset.\n")
}

# Convert columns to appropriate types
data$Chr <- as.numeric(as.character(data$Chr))  # Ensure chromosomes are numeric
data$P_BH <- as.numeric(data$P_BH)             # Ensure P_BH is numeric

# Save QQ plot as a JPEG
jpeg("qq_plot.jpeg", width = 800, height = 600)
qq(
  data$P_BH,
  main = "QQ Plot of P_BH Values",
  xlab = "Expected -log10(P)",
  ylab = "Observed -log10(P)"
)
dev.off()  # Close the JPEG device

# Save QQ plot as a PDF
pdf("qq_plot.pdf", width = 8, height = 6,
    compress = TRUE,  # This option enables PDF compression
)

qq(
  data$P_BH,
  main = "QQ Plot of P_BH Values",
  xlab = "Expected -log10(P)",
  ylab = "Observed -log10(P)"
)
dev.off()  # Close the PDF device

# Prepare data for Manhattan plot
manhattan_data <- data.frame(
  SNP = data$Name,        # SNP names
  CHR = data$Chr,         # Chromosome numbers
  BP = data$Position,     # Base pair positions
  P = data$P_BH,          # P-values
  stringsAsFactors = FALSE
)

# Sort the data by chromosome (CHR) and base pair position (BP)
manhattan_data <- manhattan_data[order(manhattan_data$CHR, manhattan_data$BP), ]

# Define custom colors for Manhattan plot
cset <- c(134, 257, 26, 76, 450, 121, 366, 630, 142, 58, 254, 548,
          542, 42, 374, 128, 116, 474, 367, 505, 29, 259, 481)
```

```
# Calculate the maximum -log10(p) value for y-axis limit
max_log_p <- max(-log10(manhattan_data$P), na.rm = TRUE)

# Extract unique chromosomes in the dataset
unique_chrs <- unique(manhattan_data$CHR)

# Save Manhattan plot as a JPEG
jpeg("manhattan_plot.jpeg", width = 1000, height = 600)
manhattan(
  manhattan_data,
  main = "Manhattan Plot",
  ylim = c(0, max_log_p + 1),                # Limit for y-axis
  genomewideline = -log10(8.216e-8),  # Threshold for genome-wide significance
  suggestiveline = FALSE,        # Disable suggestive significance line
  cex.axis = 1.1,                # Adjust axis label size
  chrlabs = as.character(unique_chrs),  # Chromosome labels
  col = colors()[cset]           # Custom colors
)
dev.off()  # Close the JPEG device

# Save Manhattan plot as a PDF
pdf("manhattan_plot.pdf", width = 10, height = 6,
    compress = TRUE,  # This option enables PDF compression
    )
manhattan(
  manhattan_data,
  main = "Manhattan Plot",
  ylim = c(0, max_log_p + 1),          # Limit for y-axis
  genomewideline = -log10(8.216e-8),  # Threshold for genome-wide significance
  suggestiveline = FALSE,        # Disable suggestive significance line
  cex.axis = 1.1,                # Adjust axis label size
  chrlabs = as.character(unique_chrs),  # Chromosome labels
  col = colors()[cset]           # Custom colors
)
dev.off()  # Close the PDF device
```

References

1. Miramontes-Gonzalez, J.P.; Usategui-Martin, R.; Martin-Vallejo, J.; Ziegler, M.; de Isla, L.L.; D, O.C.; Gonzalez-Sarmiento, R. VAV3 rs7528153 and VAV3-AS1 rs1185222 polymorphisms are associated with an increased risk of developing hypertension. *Eur J Intern Med* **2020**, *80*, 60-65, doi:10.1016/j.ejim.2020.05.014.
2. Levy, D.; Larson, M.G.; Benjamin, E.J.; Newton-Cheh, C.; Wang, T.J.; Hwang, S.J.; Vasan, R.S.; Mitchell, G.F. Framingham Heart Study 100K Project: genome-wide associations for blood pressure and arterial stiffness. *BMC Med Genet* **2007**, *8 Suppl 1*, S3, doi:10.1186/1471-2350-8-S1-S3.
3. Kulminski, A.; Feng, F.; Kulminskaya, I.; Nazarian, A.; Loiko, E.; Loika, Y. Extensive antagonistic genetic underpinnings of sex disparities in Alzheimer’s disease and hypertension. *Alzheimer's & Dementia* **2024**, e091324, doi:DOI: 10.1002/alz.091324.
4. Dollin, C.; Ward, M.; Stafford, M.Y.C.; Krason-Kidzinska, E.; Crawford, L.; McNulty, H.; Barry, F.; Murphy, M.; Lees-Murdock, D.J. Accelerated epigenetic age in hypertension: a systematic review and meta-analysis. *Hypertens Res* **2026**, doi:10.1038/s41440-025-02470-y.
5. Kamali, Z.; Keaton, J.M.; Haghjooy Javanmard, S.; International Consortium Of Blood, P.; Million Veteran, P.; e, Q.C.; Bios, C.; Edwards, T.L.; Snieder, H.; Vaez, A. Large-Scale Multi-Omics Studies Provide New Insights into Blood Pressure Regulation. *Int J Mol Sci* **2022**, *23*, doi:10.3390/ijms23147557.
6. Surendran, P.; Feofanova, E.V.; Lahrouchi, N.; Ntalla, I.; Karthikeyan, S.; Cook, J.; Chen, L.; Mifsud, B.; Yao, C.; Kraja, A.T.; et al. Discovery of rare variants associated with blood pressure regulation through meta-analysis of 1.3 million individuals. *Nat Genet* **2020**, *52*, 1314-1332, doi:10.1038/s41588-020-00713-x.
7. Tomaszewski, M.; Charchar, F.J.; Lynch, M.D.; Padmanabhan, S.; Wang, W.Y.; Miller, W.H.; Grzeszczak, W.; Maric, C.; Zukowska-Szczechowska, E.; Dominiczak, A.F. Fibroblast growth factor 1 gene and hypertension: from the quantitative trait locus to positional analysis. *Circulation* **2007**, *116*, 1915-1924, doi:10.1161/CIRCULATIONAHA.107.710293.
8. Tomaszewski, M.; Charchar, F.J.; Nelson, C.P.; Barnes, T.; Denniff, M.; Kaiser, M.; Debiec, R.; Christofidou, P.; Rafelt, S.; van der Harst, P.; et al. Pathway analysis shows association between FGFBP1 and hypertension. *J Am Soc Nephrol* **2011**, *22*, 947-955, doi:10.1681/ASN.2010080829.
9. Tomaszewski, M.; Eales, J.; Denniff, M.; Myers, S.; Chew, G.S.; Nelson, C.P.; Christofidou, P.; Desai, A.; Busst, C.; Wojnar, L.; et al. Renal Mechanisms of Association between Fibroblast Growth Factor 1 and Blood Pressure. *J Am Soc Nephrol* **2015**, *26*, 3151-3160, doi:10.1681/ASN.2014121211.
